# Splicing-dependent restriction of the HBZ gene by Tax underlies biphasic HTLV-1 infection

**DOI:** 10.1371/journal.ppat.1013381

**Published:** 2025-07-28

**Authors:** Yi Liang, Chenyang Lyu, Shuwen Xu, Chenxin Tan, Qian Jiang, Benquan Liu, Sikai Yang, Weijia Huang, Ruoning Zhou, Xiaoyi Yuan, Xiaorui Zuo, Masao Matsuoka, Guangyong Ma

**Affiliations:** 1 State Key Laboratory of Natural Medicines, China Pharmaceutical University, Nanjing, China; 2 Department of Hematology, Rheumatology and Infectious Diseases, Faculty of Life Sciences, Kumamoto University, Kumamoto, Japan; Imperial College London Faculty of Medicine, UNITED KINGDOM OF GREAT BRITAIN AND NORTHERN IRELAND

## Abstract

HTLV-1 is an oncovirus that encodes a transactivator Tax and a regulatory gene HBZ. HTLV-1 early or infectious replication depends on Tax; during HTLV-1 late infection, HBZ plays a crucial role in driving the proliferation of infected cells and maintaining viral persistence. The biphasic replication pattern of HTLV-1 dictated by Tax and HBZ represents a result of viral host adaptation, but how HTLV-1 coordinates Tax and HBZ expression to facilitate early and late infection remains elusive. Here we reveal that HBZ RNA splicing exhibits distinct patterns in Tax+ and Tax- HTLV-1 infected cells. We demonstrate that Tax interacts with the host spliceosome and inhibits HBZ splicing by competitively binding splicing factors including WDR83 and GPATCH1. As a result, Tax confers a natural constraint on HBZ, counterbalancing its anti-replication effect at HTLV-1 early infection, while unleashing HBZ to drive HTLV-1 mitotic propagation during late infection. The splicing-dependent restriction of HBZ by Tax thus represents a critical interplay central to HTLV-1 persistence.

## Author summary

HTLV-1 is an oncogenic retrovirus that exhibits a complex biphasic replication pattern governed by the transactivator Tax and the regulatory gene HBZ. This study reveals Tax as a critical splicing modulator that interacts with the host spliceosome. By inhibiting HBZ splicing through competition with host factors such as WDR83 and GPATCH1, Tax imposes a regulatory constraint on the HBZ gene, which is vital for HTLV-1 persistence. These findings enhance our understanding of the interplay between viral oncogenes in HTLV-1 infection and suggest new avenues for therapeutic intervention in oncovirus-related diseases.

## Introduction

10% of human cancers are caused by seven oncoviruses, which include hepatitis B virus (HBV), hepatitis C virus (HCV), human papillomavirus (HPV), Epstein-Barr virus (EBV), Kaposi sarcoma-associated herpesvirus (KSHV), Merkel cell polyomavirus (MCV), and human T-cell leukemia virus type 1 (HTLV-1) [1]. All oncovirus infections can enter latency *in vivo*, including HBV [2,3] and HCV [4]. The biphasic (productive and latent) replication pattern of oncoviruses represents a result of host adaptation, and oncoviruses have developed various mechanisms to coordinate distinct stages of infection. For example, KSHV encodes an early replication and transcription activator (RTA) gene required for productive infection and a latency-associated nuclear antigen (LANA) gene that promotes latency, whereby it develops a mutual antagonism between the two genes to switch between infectious and latent states [5,6]. As the first human pathogenic retrovirus identified in the 1980s, HTLV-1 currently infects 5–10 million people worldwide [7]. Approximately 10% of HTLV-1 infections lead to the development of either an aggressive malignancy known as adult T-cell leukemia (ATL) or a progressive neuroinflammatory disease called HTLV-1-associated myelopathy/tropical spastic paraparesis (HAM/TSP) [8,9]. HTLV-1 is known to enter a decades-long latency *in vivo* after early infection, yet the intrinsic mechanism governing its biphasic infection is unclear.

HTLV-1 predominantly infects CD4 T cells, integrates a reverse-transcribed viral DNA copy into chromatin, and persists lifelong in the host [10]. While HTLV-1 productive infection relies on the transactivator protein Tax, the HTLV-1 bZIP factor (HBZ) gene is indispensable for viral persistence and disease development [11–14]. HBZ is constitutively expressed in ATL cells, and its level correlates positively with viral load [15]. Furthermore, HBZ has also been shown to promote HTLV-1 infectivity in a rabbit infection model [11], highlighting its multifaceted functions during HTLV-1 early and late infection. Unlike other HTLV-1 genes, HBZ is transcribed in the antisense direction from viral 3’ long terminal repeat (LTR) located at the opposite end of the provirus [16]. The precursor HBZ RNA (HBZ pre-RNA) is approximately 4.5 kb long and is reportedly to be spliced at a conserved site to produce a mature spliced HBZ (sHBZ) RNA, which is translated into an sHBZ protein [17–19]. Interestingly, a prominent role of sHBZ is counteracting Tax functions [16]. For example, Tax activates a broad range of cellular pathways [20], yet many of these can be suppressed by sHBZ [16,21]. Several lines of evidence suggest that the functional antagonism between Tax and HBZ is a viral strategy for fine-tuning a favorable host environment [22–24]. However, this idea is challenged by the mutually exclusive expression patterns of Tax and HBZ observed *in vivo* [24]. Instead, HTLV-1 may orchestrate Tax and HBZ expression to function at the early and late stages of infection respectively, resembling other oncoviruses such as KSHV [25]. Indeed, the expression of sHBZ during early HTLV-1 infection is considered deleterious, as it suppresses viral replication [26]. On the other hand, the presence of Tax alone is detrimental to persistently infected cells *in vivo* because it induces a strong cell immunity that can eradicate the virus [27]. Therefore, HTLV-1 infection appears to benefit from the antagonistic expression of Tax and HBZ. However, the underlying mechanism remains poorly understood.

RNA splicing facilitates the modulation of specific RNA transcripts and protein isoforms [28]. Viruses frequently utilize host splicing machinery to produce spliced viral genes [29]. For example, MCV T gene is alternatively spliced to produce three distinct proteins: the large T (LT), small T (ST), and 57kT antigens, which are required at distinct stages of viral infection [30].Viruses have also evolved to dynamically regulate viral gene splicing to enhance infectivity. For instance, the influenza A virus has been reported to utilize suboptimal splicing to generate inefficiently spliced viral transcripts, in order to coordinate the timing of infection [31]. Here, we characterize for the first time that HTLV-1 transactivator Tax directly restricts the splicing and expression of the HBZ gene. The critical interplay between HTLV-1 Tax and HBZ represents a previously unknown intrinsic mechanism by which HTLV-1 coordinates productive and latent infection for lifelong persistence *in vivo*.

## Results

### HBZ exhibits distinct splicing patterns in Tax+ and Tax- HTLV-1 infected cells

**Fig 1:**
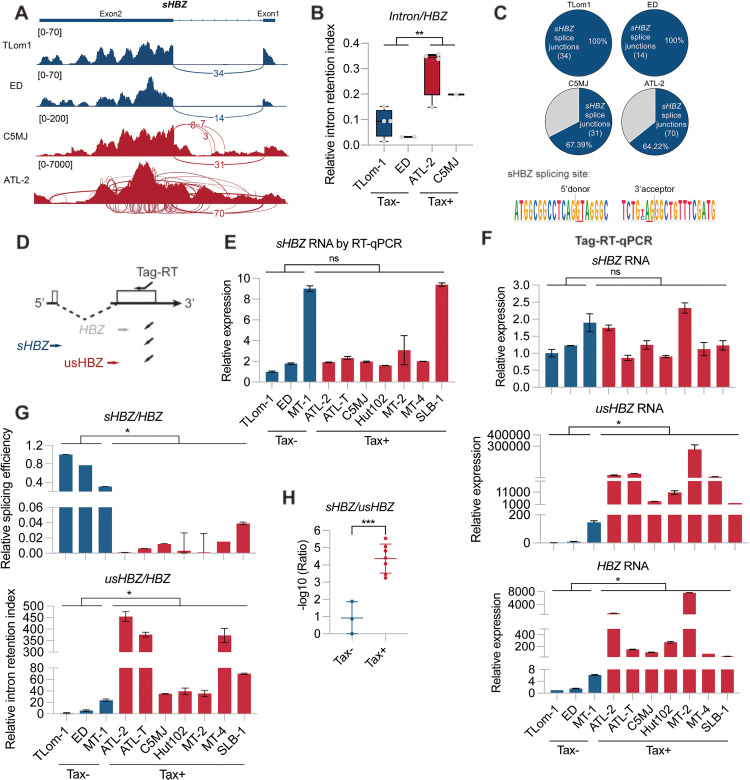
HBZ exhibits distinct splicing patterns in Tax + and Tax- HTLV-1 infected cells. (A) Sashimi plot of mapped HBZ RNA reads in representative HTLV-1 infected T cell lines, including Tax-negative TL-Om1 and ED, and Tax-positive C5MJ and ATL-2. RNA-seq data were obtained from the ENA database (https://www.ebi.ac.uk/ena). Sample accession IDs are included in S6 Table. (B) The relative HBZ intron retention index in HTLV-1 infected T cells, calculated by normalizing the number of HBZ intron reads to total HBZ reads. (C) (*Upper*) Pie chart showing the percentages of sHBZ splice junctions (SJ) and others in total HBZ SJs. (*Bottom*) The Logo plot indicates the sHBZ splice site is conserved in 134 HTLV-1 strains. (D) Schematic diagram of locations of primers for Tag-RT-qPCR. (E) Relative expression of sHBZ in HTLV-1 infected T cells assessed by RT-qPCR. ACTB was used as the internal control for normalization. (F) Relative expression of (*Upper*) sHBZ, (*Middle*) usHBZ, and (*Bottom*) HBZ in HTLV-1 infected T cells assessed by Tag-RT-qPCR. ACTB was used as the internal control for normalization. (G) (*Upper*) Relative splicing efficiency (sHBZ/HBZ) and (*Bottom*) relative intron retention index (usHBZ/HBZ) of HBZ in HTLV-1 infected T cells. (H) Ratio of relative expression of sHBZ to usHBZ in Tax- (Blue) and Tax+ (Red) HTLV-1-infected T cells. *P* value was calculated using a two-tailed Student’s t-test. ns, not significant; ^*^P < 0.05, ^**^P < 0.01, ^***^P < 0.001. The results are representatives of two independent experiments.

As an indispensable regulatory gene of HTLV-1, the RNA splicing of HBZ has never been fully investigated. By analyzing the strand-specific RNA-seq results of HTLV-1 infected cell lines [32–35], we discovered that the endogenous HBZ RNA in Tax+ and Tax- cells exhibits distinct splicing patterns (Fig 1A). While HBZ splicing was efficient in Tax- cells, it was not in Tax+ cells and had increased occurrence of intron retention (Figs 1A, 1B and S1A). In addition, multiple novel splice junctions of HBZ were detected in Tax+ cells (Fig 1C), indicating that the retained HBZ introns were likely alternatively spliced. To further quantify the efficiency of HBZ splicing, we performed a strand-specific Tag-RT-qPCR assay [36], in which a Tag-containing primer initiated the reverse transcription (Fig 1D). The result of Tag-RT-qPCR was similar to that of conventional RT-qPCR, suggesting the validity of this assay (Fig 1E and 1F). It should be noted that the primer specificity in both Tag-RT-qPCR and RT-qPCR assays has been validated (S1B Fig). Although sHBZ was expressed at comparable levels in Tax- and Tax+ cells, unspliced HBZ (usHBZ) [37] and the HBZ pre-RNA were much more abundant in Tax+ cells (Fig 1F). The efficiency of HBZ splicing, quantified as a ratio of sHBZ to HBZ pre-RNA level, was significantly higher in Tax- cells, while their HBZ intron retention rate (usHBZ to HBZ pre-RNA level) was lower (Fig 1G). We hereby uncovered a surprising finding that Tax expression is invariably associated with impaired HBZ splicing in HTLV-1 infected cells, resulting in a significantly reduced sHBZ/usHBZ ratio (Fig 1H).

### Endogenous Tax suppresses HBZ RNA splicing in HTLV-1 infected cells

**Fig 2:**
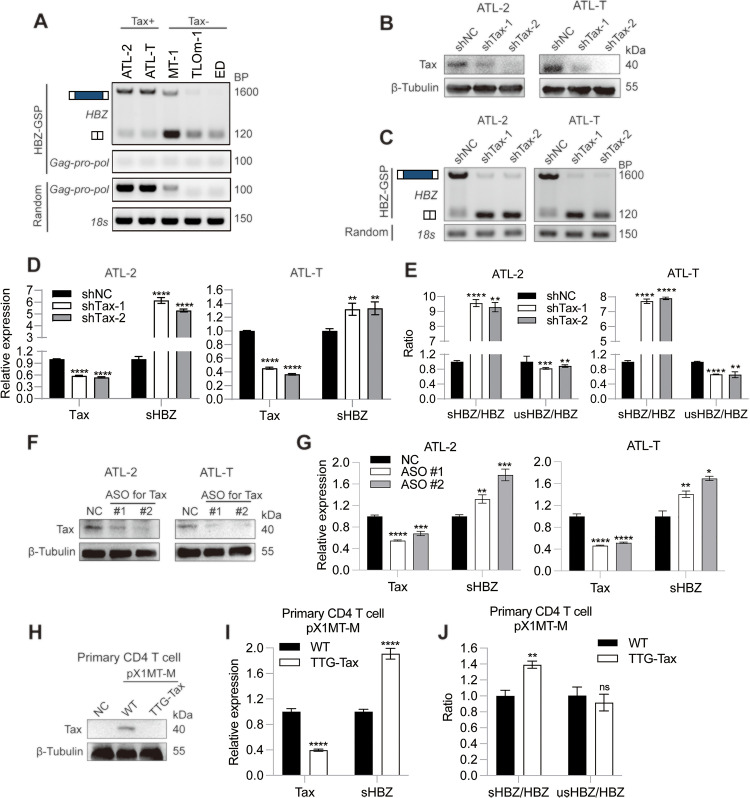
Endogenous Tax suppresses HBZ RNA splicing in HTLV-1 infected cells. (A) RT-PCR of target genes from the HBZ-gene specific primer (GSP) library and random primer library in HTLV-1 infected T cells. (B) Immunoblot result verifying the shRNA-mediated knockdown of Tax in ATL-2 and ATL-T. (C) RT-PCR analysis of HBZ RNA splicing following shRNA-mediated knockdown of Tax. (D) qPCR analysis of Tax and sHBZ expression levels following shRNA-mediated knockdown of Tax. (E) Tag-RT-qPCR analysis of the ratios of sHBZ/HBZ and usHBZ/HBZ following shRNA-mediated knockdown of Tax. (F) Immunoblot result verifying the ASO-mediated knockdown of Tax. (G) qPCR analysis of Tax and sHBZ expression levels following ASO-mediated knockdown of Tax. (H) Immunoblot analysis of Tax protein expression in primary CD4 T cells *de novo* infected with HTLV-1 infectious clones pX1MT-M-WT or pX1MT-M-TTG-Tax. (I) qPCR analysis of Tax and sHBZ expression levels in infected primary CD4 T cells. (J) Tag-RT-qPCR analysis of the ratios of sHBZ/HBZ and usHBZ/HBZ in infected primary CD4 T cells. *P* value was calculated using a two-tailed unpaired Student’s t-test. ns, not significant; ^*^P < 0.05, ^**^P < 0.01, ^***^P < 0.001, ^****^P < 0.0001. The results are representatives of three independent experiments.

By using an HBZ-specific RT primer, we performed RT-PCR and confirmed the accumulation of the unspliced HBZ pre-RNA in Tax+ but not Tax- HTLV-1 infected cells (Fig 2A), agreeing with the results of Tag-RT-qPCR (Fig 1). To determine whether Tax regulates HBZ splicing *in vivo*, we knocked down Tax in HTLV-1 infected ATL-2 and ATL-T cells (Fig 2B). HBZ-specific RT-PCR demonstrated that Tax depletion resulted in an increased sHBZ but decreased HBZ pre-RNA expression (Fig 2C), indicating that there was persistent restriction on HBZ RNA splicing by endogenous Tax. Consistently, RT-qPCR verified the concomitant increase of sHBZ RNA level upon Tax depletion (Fig 2D), linking Tax to the repressed HBZ splicing and increased intron retention *in vivo* (Fig 2E). Antisense oligonucleotide (ASO) mediated Tax knockdown, which is expected to effectively prevent off target effect, also confirmed the above results (Fig 2F and 2G). Moreover, we verified this function of Tax in HTLV-1 *de novo* infected human CD4 T cells. By using a TTG-Tax mutant of HTLV-1 infectious clone pX1MT-M (Fig 2H), we demonstrated that knocking out Tax protein increased sHBZ expression (Fig 2I) and the splicing efficiency of HBZ RNA (Fig 2J) during HTLV-1 infection. Therefore, Tax is able to inhibit HBZ splicing and sHBZ RNA expression at the endogenous level, imposing a persistent restriction on the HBZ gene.

### Tax directly inhibits HBZ splicing in a transactivation-independent manner

**Fig 3:**
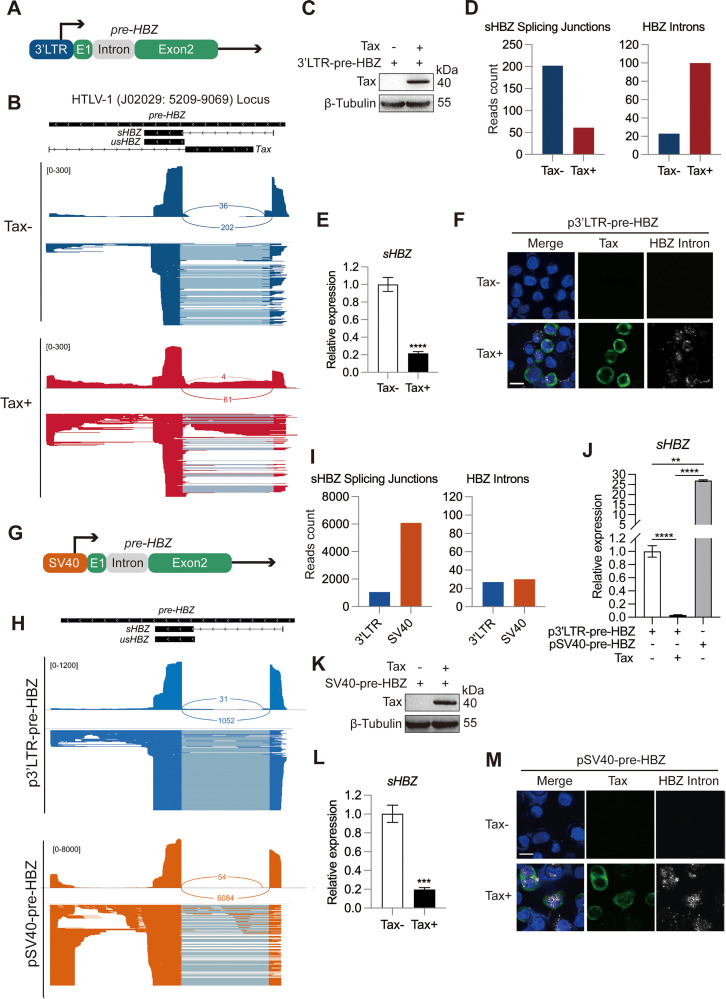
Tax directly inhibits HBZ RNA splicing in a transactivation-independent manner. (A) Schematic diagram of the plasmid p3’LTR-pre-HBZ. (B) Sashimi plot of HBZ expressed by p3’LTR-pre-HBZ in HeLa S3 cells under Tax- or Tax+ conditions, generated by ONT cDNA-seq. (C) Immunoblot result showing the expression of Tax. (D) Bar chart statistics of (*Left*) sHBZ SJs and (*Right*) HBZ introns reads in (B). (E) qPCR result showing relative abundance of sHBZ expressed by p3’LTR-pre-HBZ, in the presence or absence of Tax. (F) RNA-FISH result showing the expression of Tax (green) and HBZ intron (white). Scale bar, 10 μm. (G) Diagram of the plasmid composition of pSV40-pre-HBZ. (H) Examination of the effect of SV40 promoter on HBZ pre-RNA splicing using ONT cDNA-seq. For (B) and (H), the upward alignment represents the coverage and splicing junctions, while the downward alignment shows the distribution of reads. (I) Bar chart statistics of (*Left*) sHBZ SJs and (*Right*) HBZ introns reads in (H). (J) qPCR analysis of the relative levels of sHBZ expressed by HBZ pre-RNA in different experimental settings. (K) Immunoblot result showing Tax expression. (L) qPCR analysis of the effect of Tax on sHBZ expression in SV40-pre-HBZ. (M) RNA-FISH images showing the expression levels of Tax and HBZ intron (scale bar, 10 μm). *P* value was calculated using a two-tailed unpaired Student’s t-test. ^**^P < 0.01, ^***^P < 0.001, ^****^P < 0.0001. The results are representatives of three independent experiments.

To investigate whether Tax directly inhibits HBZ RNA splicing, we cloned the native HBZ expression cassette from the HTLV-1 provirus, which includes a 3’ LTR promoter and a complete HBZ pre-RNA sequence (4.5 kb) (Fig 3A), into a vector from which the original promoter had been removed. Then we transfected this construct (p3’LTR-pre-HBZ) into HeLa S3 cells and performed Oxford Nanopore Technologies (ONT) cDNA-seq [38] to capture the full-length HBZ transcripts. An sHBZ-dominant transcription pattern resembling that of the endogenous HBZ in Tax- cells was observed (Fig 3B), suggesting the efficient splicing of the HBZ pre-RNA. However, co-expression of Tax markedly suppressed HBZ splicing (Fig 3B and 3C), as demonstrated by the reduced number of sHBZ splice junctions reads and the increased number of HBZ intron reads (Fig 3D). Consequently, the abundance of sHBZ RNA was downregulated by Tax (Fig 3E). As verification, Tax-induced HBZ intron retention was visualized by single molecule RNA-fluorescence in situ hybridization (RNA-FISH) [39] (Fig 3F).

RNA splicing is coupled with transcription and is therefore subject to the regulation by promoter [40]. Since Tax was found to augment the activity of HBZ promoter (S2 Fig), we asked if increased promoter activity accounts for the suppression on HBZ RNA splicing. Contrary to our hypothesis, HBZ splicing (Fig 3G-3I) and sHBZ expression (Fig 3J) were all instead enhanced when HBZ was expressed by a stronger SV40 promoter (pSV40-pre-HBZ). Strikingly, co-expression of Tax still effectively inhibited the RNA splicing of HBZ expressed by pSV40-pre-HBZ (Fig 3K-3M), demonstrating the strong ability of Tax in repressing HBZ splicing. These results collectively imply that the splicing modulator role of Tax is independent from its transactivation function.

### Proximity labelling identifies Tax interacts with the host spliceosome

**Fig 4:**
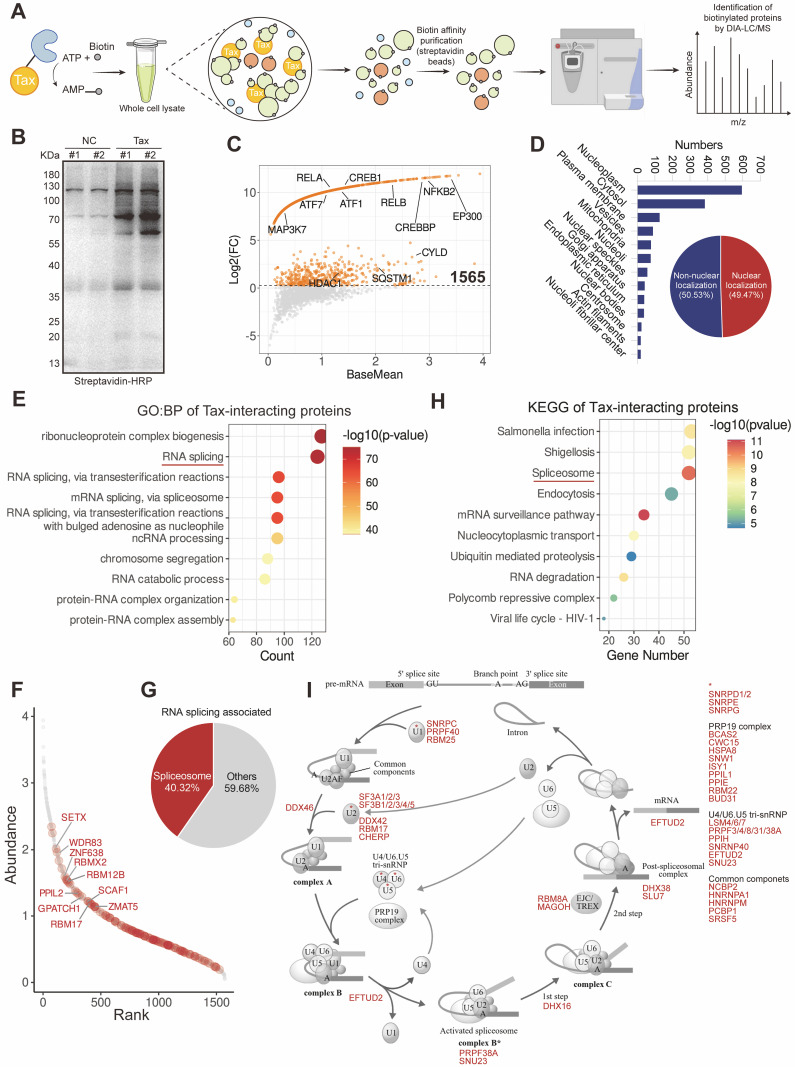
Proximity labelling identifies HTLV-1 Tax interacts with the host spliceosome. (A) Schematic depicting the miniTurboID workflow for identifying Tax interacting proteins. The diagram was drawn by hand using Adobe Illustrator (https://www.adobe.com/products/illustrator.html). (B) Immunoblot analysis demonstrating the biotinylated proteins pulled down from the samples. (C) Scatter plots showing the fold change (FC) (log2-FC) and signal intensity (BaseMean) of proximity-labeled proteins, with a FC greater than 1.2 considered positive interaction. Known Tax-interacting proteins are marked. n = 2 biological replicates for Tax-miniTurboID and NC-miniTurboID samples. The scores of proteins that were not detected in the NC group (hence 0) were set to 0.001 to facilitate FC calculation. The proteins concentrated in the upper curve in the figure represent those with high specificity for binding to Tax. (D) The subcellular localization of Tax-interacting proteins in the Cell Atlas database [47] (https://www.proteinatlas.org/). (*Upper*) The bar chart showing all subcellular localizations. (*Bottom*) The pie chart displaying the percentages of non-nuclear and nuclear proteins. (E) The top 10 entries for GO:BP enrichment analysis of Tax-interacting proteins. (F) Abundance ranking of Tax-interacting proteins, with red dots indicating RNA splicing-related proteins. The top 10 proteins based on FC are highlighted. (G) Pie chart displaying the proportion of spliceosome components among RNA splicing-related proteins in (E). (H) The top 10 entries for KEGG enrichment analysis of Tax-interacting proteins. (I) Tax interacting proteins (marked in red) are components of the spliceosome, as revealed by the KEGG spliceosome pathway (map03040).

To test if Tax may compete with splicing factors responsible for HBZ RNA splicing, we tried to obtain the Tax-interactome first. Proximity labeling has emerged as a novel and powerful technique to capture protein interactions in native settings, particularly transient and weak interactions that are difficult to detect using conventional co-immunoprecipitation (Co-IP) [41,42]. So we performed miniTurboID-based proximity labeling of Tax in HeLa S3 cells (Figs 4A, 4B and S3), and identified 1,565 high-confidence candidate Tax-interacting proteins (Fig 4C and S1 Table), including known Tax-binding transcription factors such as EP300 [43], CREB1 [44], and RELA [45] (Fig 4C). Both nuclear and non-nuclear proteins were identified (Fig 4D), which agrees with the subcellular localization pattern of Tax [46,47]. In line with the role of Tax in splicing control, Gene Ontology (GO) analysis revealed significant enrichment of Tax-binding proteins in RNA-associated pathways, especially RNA splicing (Fig 4E and S2 Table). Further examination of Tax-binding proteins enriched in the above entry (Fig 4F) suggested that over 40% of them were components of spliceosomal complexes (Fig 4G). Kyoto Encyclopedia of Genes and Genomes (KEGG) analysis also identified an association between Tax and the spliceosome (Fig 4H and S3 Table). Importantly, Tax-interacting proteins were widely present in various subcomplexes of the spliceosome that are required at different stages of splicing (Fig 4I), suggesting that Tax may impact the entire RNA splicing process.

### Tax targets spliceosomal proteins that interact with HBZ pre-RNA

**Fig 5:**
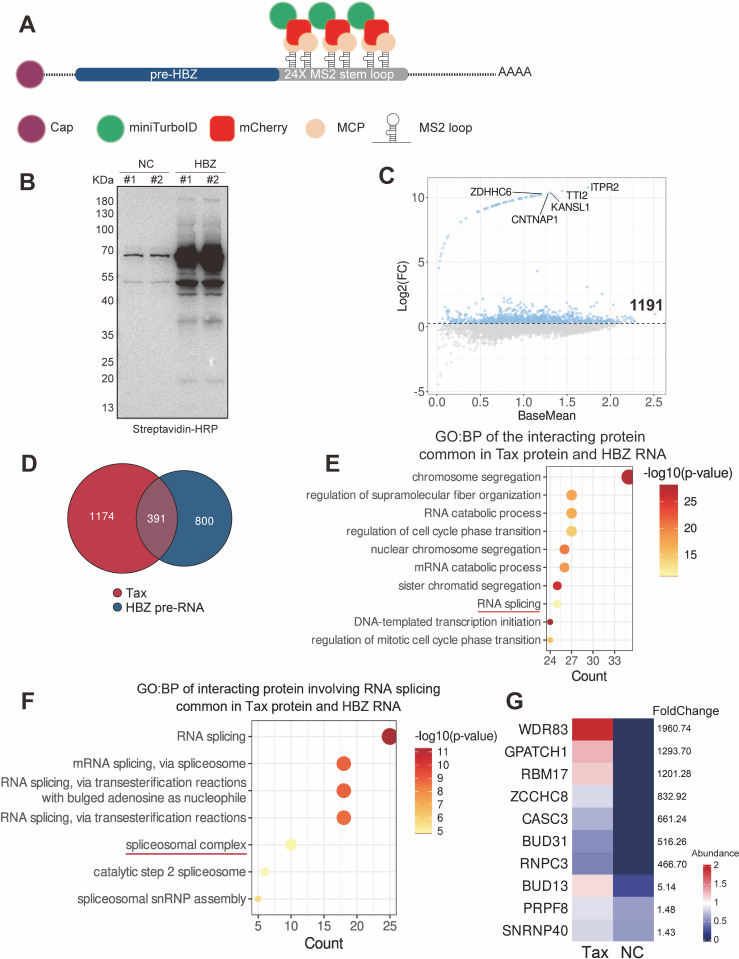
Tax targets spliceosomal proteins that interact with HBZ pre-RNA. (A) Diagram illustrating the principle of the RNA-bioID method [48]. (B) Immunoblot result demonstrating the biotinylated proteins pulled down from the samples. (C) Scatter plots showing the FC (log2-FC) and signal intensity (BaseMean) of proximity-labeled proteins. FC > 1.2 is considered positive (in blue). The names of top 5 proteins based on FC are marked. n = 2 biological replicates for HBZ pre-RNA and NC samples. The scores of proteins that were not detected in the NC group (hence 0) were set to 0.001 to facilitate FC calculation. The proteins concentrated in the upper curve in the figure represent those with high specificity for binding to HBZ RNA. (D) Intersection of Tax and HBZ pre-RNA interacting proteins. (E) Top 10 entries from the GO: BP enrichment analysis of the 391 intersected proteins. (F) GO: BP enrichment analysis of the common interacting proteins of Tax and HBZ pre-RNA enriched by the RNA splicing entry. (G) The abundance and FC of proteins in the spliceosomal complex shown in (F).

Next we set to screen for host splicing factors associated with HBZ pre-RNA. RNA proximity labelling (RNA-bioID) combines a biotin ligase with endogenously MS2-tagged RNA to achieve proximity biotinylation of RNA interacting proteins, thereby identifying new RNA interactors (Fig 5A). As Tax interacts with a number of splicing factors (Fig 4), to pinpoint the one(s) mediating HBZ splicing, we performed proximity labeling of HBZ pre-RNA [48] (S4 Fig). We identified a total of 1,191 candidate interacting proteins (Figs 5B, 5C and S4 Table), of which 391 proteins can interact with both Tax protein and HBZ pre-RNA (Fig 5D). By annotating the above proteins, we discovered that 25 of them were associated with RNA splicing (Fig 5E). Since Tax interacts with the spliceosome, we then focused on a spliceosomal complex entry (Fig 5F) comprising 10 proteins (Fig 5G). Among these 10 candidates, WD repeat domain 83 (WDR83) and G-patch domain containing 1 (GPATCH1) attracted our attention (Fig 5G). WDR83 is a subunit of spliceosome complexes C and P while GPATCH1 is a subunit of spliceosome complex P. Complex C catalyzes the removal of introns, whereas the subsequent release of mRNA is mediated by complex P [49]. Based on their higher abundances (Fig 5G) and essential roles in RNA splicing, we selected WDR83 and GPATCH1 for further investigation.

### Tax impairs HBZ RNA splicing via binding WDR83 and GPATCH1

**Fig 6:**
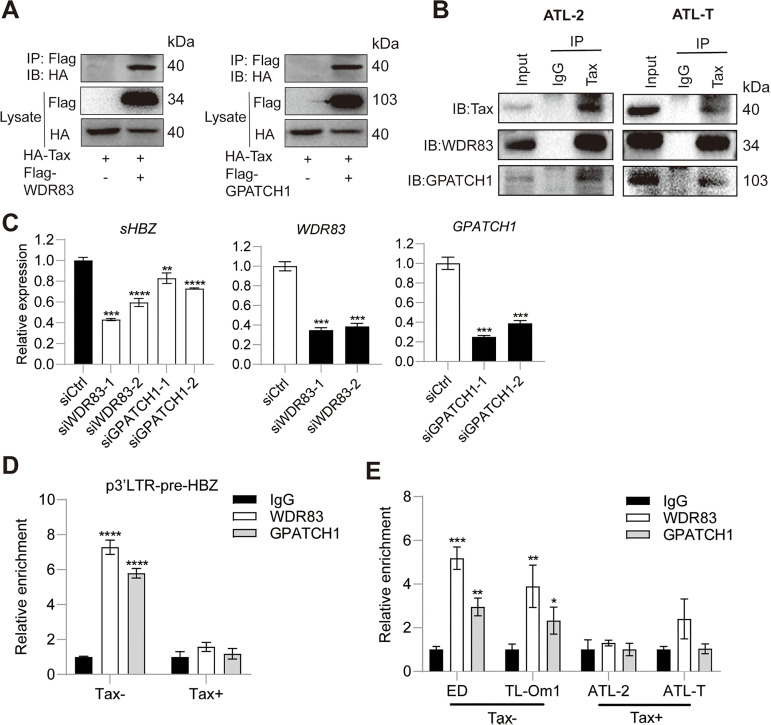
Tax impairs HBZ RNA splicing via binding WDR83 and GPATCH1. (A) Co-IP results of HA-Tax with Flag-WDR83 or Flag-GPATCH1 in HeLa S3 cells. (B) Co-IP results of Tax with WDR83 or GPATCH1 at endogenous levels in ATL-2 and ATL-T. (C) qPCR analysis of the impact of WDR83 and GPATCH1 knockdown on sHBZ expressed by p3’LTR-pre-HBZ in HeLa S3. (D) RIP-qPCR result demonstrating Tax impairs the binding of WDR83 or GPATCH1 with HBZ RNA in HeLa S3. (E) RIP-qPCR analysis demonstrating impaired binding of WDR83 or GPATCH1 with HBZ RNA in Tax+ but not Tax- HTLV-1 infected T cells. *P* value was calculated using a two-tailed unpaired Student’s t-test. ^*^P < 0.05, ^**^P < 0.01, ^***^P < 0.001, ^****^P < 0.0001. The results are representatives of three independent experiments.

In order to examine the possible interaction between Tax and WDR83 or GPATCH1, we co-expressed these proteins in HeLa S3 cells and performed Co-IP. As shown in Fig 6A, physical association was confirmed between Tax and WDR83 or GPATCH1. Furthermore, Tax also interacted with WDR83 or GPATCH1 at endogenous levels in HTLV-1 infected ATL-2 and ATL-T cells (Fig 6B). Importantly, knockdown of WDR83 or GPATCH1resulted in the downregulation of sHBZ RNA level (Fig 6C), suggesting a positive role for both proteins in mediating HBZ splicing. Both splicing factors were demonstrated by RNA immunoprecipitation to be recruited to HBZ RNA (Fig 6D); however, their recruitments were markedly suppressed in the presence of Tax (Fig 6D). In addition, the association of endogenous WDR83 or GPATCH1 and HBZ RNA was only observed in Tax- but not Tax+ HTLV-1 infected cells, highlighting the potential interference of Tax on the recruitment of WDR83 or GPATCH1 to HBZ RNA (Fig 6E). Collectively, we demonstrated that Tax is able to directly interfere with HBZ RNA splicing via competitively binding WDR83 and GPATCH1.

### Tax-mediated sHBZ restriction impacts HTLV-1 infection and persistence

**Fig 7:**
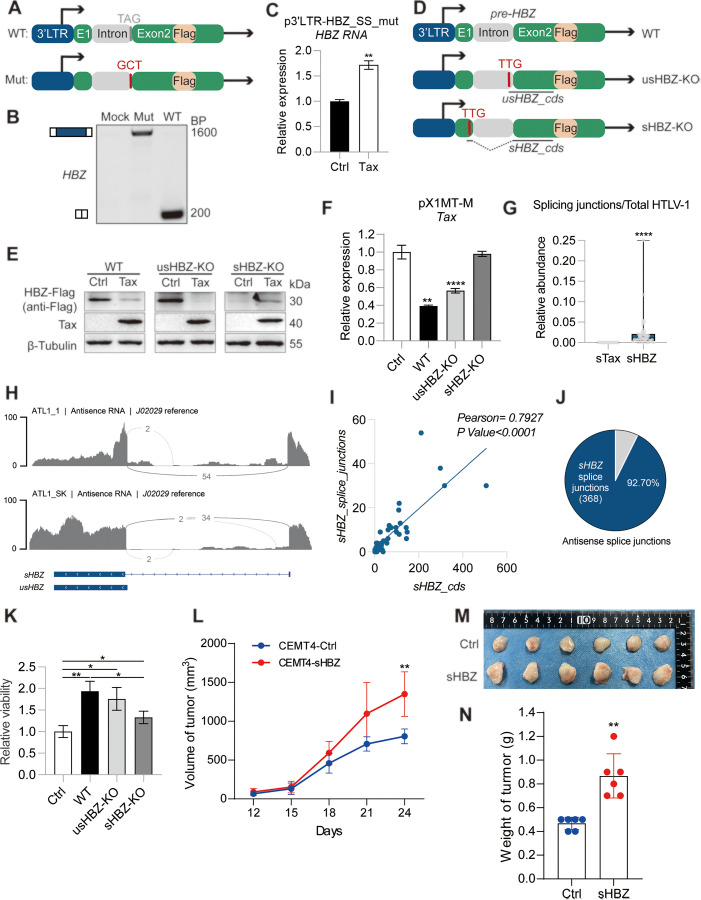
Tax mediated sHBZ restriction impacts HTLV-1 infection and persistence. (A) Schematic diagrams of wild-type (WT) and the 3’ acceptor splice site mutant (Mut) of p3’LTR-pre-HBZ. (B) RT-PCR analysis demonstrates that HBZ RNA fails to undergo splicing following mutation of the 3’ splice site. (C) qPCR analysis indicates Tax enhances the transcription of HBZ RNA expressed by the p3’LTR-pre-HBZ 3’ acceptor splice site mutant. (D) Schematic diagrams of the p3’LTR-pre-HBZ-Flag plasmids with the native HBZ sequence (WT) or having usHBZ or sHBZ start codons mutated to TTG to knock out usHBZ or sHBZ protein, respectively. (E) Immunoblot result showing the effect of Tax on the expression of Flag-HBZ proteins. (F) qPCR analysis of the effect of WT, sHBZ-KO, or usHBZ-KO p3’LTR-pre-HBZ on the expression of Tax in HeLa S3 cells transfected with the HTLV-1 infectious clone pX1MT-M. (G) Relative abundances of sTax (spliced Tax) and sHBZ by normalizing respective splice junction reads to total HTLV-1 reads in ATL patients (PRJEB19394). (H) Sashimi plot of HBZ RNA of representative ATL patients. (I) The correlation between sHBZ CDS and splice junction reads in ATL patients analyzed using Pearson’s method. (J) sHBZ is the dominant isoform of HBZ in ATL patients. (K) Effects of WT, sHBZ-KO, or usHBZ-KO p3’LTR-pre-HBZ on the proliferation of CEMT4 cells. (L) Tumor growth curve of xenograft mice established by injecting CEMT lines stably expressing sHBZ or empty control. (M) Images of tumors derived from CEMT4-Ctrl or CEMT4-sHBZ injected xenograft mice, photographed by us. n = 6. (N) Tumor weights in (M). *P* value was calculated using a two-tailed Student’s t-test. ^*^P < 0.05, ^**^P < 0.01, ^****^P < 0.0001. The results (except xenograft) are representatives of three independent experiments.

We have presented substantial evidence that Tax mediates sHBZ restriction via interfering with its RNA splicing. The fact that disrupting the splicing site of sHBZ (Fig 7A and 7B) completely abolished the restriction of HBZ by Tax (Fig 7C) further supports the splicing-dependent nature of such regulation. To prove Tax suppresses sHBZ protein expression via splicing, we added an in-frame Flag tag right after HBZ coding sequence (CDS) in the HBZ pre-RNA expression vector (p3’LTR-pre-HBZ) (Fig 7D) and performed an immunoblot. We successfully detected the Flag-tagged sHBZ protein, which was markedly suppressed by Tax as expected (Fig 7E). By silencing the translation of sHBZ or usHBZ, respectively (Fig 7D), we showed that only sHBZ protein expression was affected by Tax (Fig 7E), consistent with the splicing modulation role of Tax. sHBZ is known to inhibit the activity of HTLV-1 5’LTR, the promoter of Tax, and we here confirmed that p3’LTR-pre-HBZ that mainly produces an sHBZ protein (Fig 7E) is able to inhibit Tax RNA expressed by pX1MT-M as well. However, knocking out sHBZ, but not usHBZ, completely abolished such inhibition (Fig 7F). Considering Tax drives HTLV-1 replication, mediating the sHBZ restriction is likely a beneficial strategy of Tax to promote viral replication. On the contrary, at late or leukemic stage of HTLV-1 infection, Tax is rarely expressed, rendering its restriction on HBZ incompetent. In line with this notion, Tax deficiency was widely observed in ATL patients whereas sHBZ was ubiquitously expressed (Fig 7G). We analyzed the strand-specific RNA-seq data of 43 ATL patients [50], and found that the reads covering the splicing junction of Tax (sTax) were completely undetectable in all ATL patients, suggesting the lack of Tax expression (Fig 7G). In contrast, the reads covering the splicing junction of HBZ (sHBZ) were detected in most (37/43) ATL patients, indicating that sHBZ is widely expressed (Fig 7G). Notably, HBZ RNA was efficiently spliced *in vivo* (Fig 7H) with sHBZ being the dominant isoform (Fig 7I and 7J), which not only connects the Tax deficiency to efficient HBZ splicing in ATL patients, but also highlights the importance of sHBZ. Indeed, we demonstrated that transfection of p3’LTR-pre-HBZ was able to enhance the proliferation of a human CD4 T cell line CEMT4, while knocking out sHBZ protein greatly impaired such effect (Fig 7K). By generating a CEMT4 line stably expressing sHBZ (S5A Fig), we proved for the first time that sHBZ alone promoted human T cell growth *in vitro* independent from a co-signal such as interleukin-2 (S5B Fig). Mechanistically, sHBZ expression was associated with accelerated cell cycle progression and reduced apoptosis (S5C–S5G Fig). In particular, sHBZ was found to promote CEMT4-mediated tumor development in a xenograft mouse model (Fig 7L–7N). Therefore, the acquired Tax deficiency during HTLV-1 persistent infection is assumed to unleash sHBZ to sustain the survival and even promote oncogenesis of infected T cells.

## Discussion

**Fig 8:**
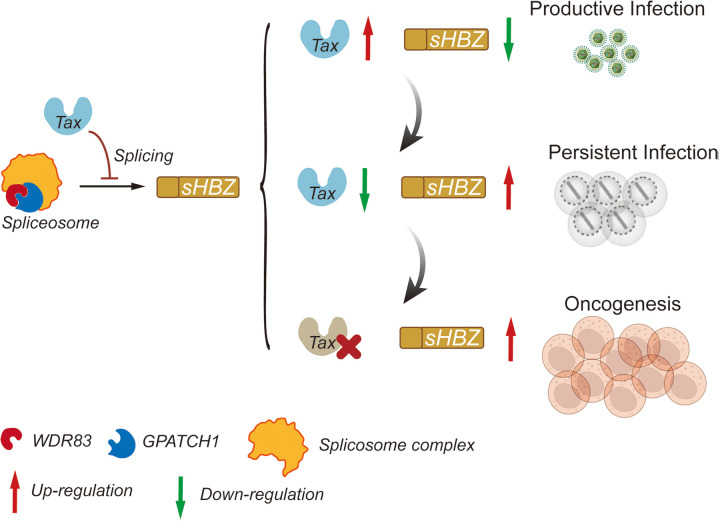
A proposed model for HTLV-1 biphasic replication based on the splicing-dependent restriction of the HBZ gene by Tax. The diagram was drawn by hand using Adobe Illustrator (https://www.adobe.com/products/illustrator.html).

Viruses frequently employ the host splicing machinery to achieve diversified gene expression, particularly those with compact genomes. MCV genome is only 5.4 kb long, and its oncogenic T gene relies on splicing to generate distinct T antigens [51]. Interestingly, LT, an oncoprotein that activates MCV replication, is frequently mutated in tumor cells [51], similar to HTLV-1 Tax. In contrast, another MCV oncoprotein, ST, is constantly expressed in tumor cells and counteracts LT function [51], resembling HTLV-1 HBZ. Therefore, the functional antagonism between viral transactivators and latent oncogenes may represent a common mechanism among oncoviruses for sustaining viral persistence. Here we report for the first time that HTLV-1 programs an intrinsic restriction of the HBZ gene by the transactivator protein Tax to coordinate productive and persistent infection, greatly furthering our understanding of oncoviruses. Notably, Tax-mediated HBZ restriction is dependent on RNA splicing, representing a unique mechanism of HTLV-1 persistence that is not found in other oncoviruses. During early HTLV-1 infection, Tax likely counteracts the potential inhibition of viral replication by sHBZ via preventing efficient HBZ RNA splicing (Fig 8). Whereas during HTLV-1 persistent infection *in vivo*, particularly when Tax expression decreases or disappears, sHBZ is likely expressed and promotes the proliferation of infected cells, sustaining the infection or leading to potential leukemogenesis (Fig 8).

Tax has been extensively studied as a causative factor for ATL; however, its expression is frequently lost in ATL due to epigenetic and genetic ablations in Tax gene or its promoter, 5’ LTR [20]. Given that Tax is a major target of the host immune system, its contribution to *in vivo* HTLV-1 persistence has been debated. Intriguingly, muted Tax expression has been shown to be reversible or inducible in some cases [52–54]. Moreover, recent findings revealed that Tax can be intermittently expressed in HTLV-1 infected cells, indicating the possibility of transient viral reactivation [55]. Our findings that Tax acts as a critical splicing modulator of HBZ reestablish its central role in HTLV-1 persistence and pathogenesis.

Recent publications have investigated the role of Tax in the regulation of host gene splicing [56,57]. In this study, we characterized Tax as a crucial regulator of viral RNA splicing. We demonstrated that Tax interacts with spliceosome components WDR83 and GPATCH1, reducing their binding affinity for HBZ RNA. By hijacking core components of the host spliceosome, Tax is expected to also impact host splicing landscape and cellular functions. Interestingly, sHBZ has also been shown to regulate global RNA splicing, yet it remains to be determined whether it associates with the host spliceosome like Tax. Nevertheless, the convergence of Tax and HBZ in modulating RNA splicing underscores the importance of splicing control in viral persistence.

It is known that among HTLV-1 infected cell lines, those expressing Tax normally have multiple copies of provirus integrated into the chromatin; in contrast, those lacking Tax expression often contain only one copy of integrated provirus. One would expect that more HTLV-1 proviral DNA loads would lead to more viral mRNA copies produced. Indeed, stranded RNA-seq results clearly show that there are much more HBZ reads in Tax+ than in Tax- cells (Fig 1A). A positive correlation was also observed between HTLV-1 proviral loads and the abundances of HBZ transcripts (S1C Fig). On the other hand, more HTLV-1 proviral loads often leads to more Tax expression. Considering that Tax restricts sHBZ expression by inhibiting HBZ RNA splicing, even though Tax+ cells harbor more HBZ transcripts, their sHBZ abundance is suppressed by Tax to a level comparable to that of Tax- cells (Fig 1E). This study therefore answers a long-standing question in the field, which is why sHBZ expression is not significantly higher in Tax+ cells in spite of more proviral DNA copies in these cells (Fig 1E). sHBZ expression is maintained at a relatively stable level across different HTLV-1 infected cell lines, irrespective of Tax expression. This fine-tuning of sHBZ abundance may imply the importance of a previously unrecognized expression control of the HBZ gene. On the other hand, it also highlights the necessity of the negative regulation of sHBZ expression by Tax.

Overall, this study characterizes HTLV-1 Tax as a critical splicing modulator that interacts with host spliceosomal components to regulate RNA splicing of viral oncogene HBZ. By inhibiting HBZ splicing through competition with host splicing factors, Tax imposes a regulatory constraint on the HBZ oncogene, which is vital for HTLV-1 persistence. However, this study has its limitations. First, most of our results were obtained using HTLV-1 infected cell lines. Therefore, it is important in the future study to use primary HTLV-1 infected cells to confirm our findings. Second, although we validated the specificity of our PCR assays (S1B Fig), it should be noted that amplification bias may occur and cause overrepresentation of HBZ, such as the unusually high number of HBZ transcripts in Tax+ HTLV-1 infected cell lines (Fig 1F). Nevertheless, our findings enhance the current understanding of the interplay between viral and host factors in oncovirus infection, and may suggest new avenues for therapeutic intervention in oncovirus-related diseases.

## Materials and methods

### Ethics statement

CD4 microbeads (Miltenyi Biotec) were used to isolate CD4 T cells from the peripheral blood mononuclear cells of a healthy human donor, from whom a written formal consent was obtained. The experiments using primary samples in this study were conducted according to the principles expressed in the Declaration of Helsinki and approved by the Institutional Review Board of China Pharmaceutical University.

### Cell lines

HTLV-1 infected cell lines, including Tax-negative ED, TLom-1, MT-1, and Tax-positive ATL-2, ATL-T, C5MJ, Hut102, MT-2, MT-4, SLB-1 were cultured in RPMI supplemented with 10% fetal bovine serum (FBS). The HTLV-1 uninfected T cell lines, CEMT4-Ctrl and CEMT4-HBZ, were also cultured in RPMI with 10% FBS. HeLa S3 and HEK293T cells were maintained in DMEM supplemented with 10% FBS. All cell lines were kept in a humidified incubator at 37 °C with 5% CO₂.

### Plasmid construction

To construct p3’LTR-pre-HBZ, we cloned the 3’ LTR-pre-HBZ sequence from the HTLV-1 infectious clone pX1MT-M into pME18S, and replaced the original SRα promoter with a SV40 poly(A) signal as an insulator. For the construction of pSV40-pre-HBZ, the 3’LTR sequence of p3’LTR-pre-HBZ was replaced with the SV40 promoter.

To detect the HBZ protein, we added a Flag tag to the C-terminus of HBZ CDS in p3’LTR-pre-HBZ. To differentiate between sHBZ and usHBZ, we mutated the start codon of sHBZ or usHBZ in p3’LTR-pre-HBZ-FLAG to TTG, allowing for the detection of usHBZ or sHBZ protein, respectively.

For knockdown (KD) experiments, we generated lentiviral vectors (pLKO.1-puro) expressing shRNAs targeting the Tax CDS (shTax1: gccttcctcaccaatgttc or shTax2: ggcagatgacaatgaccatga) or a negative control (shNC: caacaagatgaagagcaccaa).

For proximity labelling, the CDS of Tax was cloned into the pCMV-miniTurboID-Flag vector (Beyotime, China). For the HBZ RNA-miniTurboID assay, the CMV-24X MS2 stem loop, synthesized by GeneScript (China), was used to replace the SRα promoter in the pME18S vector to generate pCMV-MS2. The pre-HBZ sequence was cloned upstream of MS2 in pCMV-MS2 to generate pCMV-pre-HBZ-MS2. CMV-NLS-MCP-mCherry-miniTurboID was synthesized by GeneScript and used to replace the SRα promoter in pME18S to create pCMV-NLS-MCP-mCherry-miniTurboID.

To construct an HTLV-1 infectious clone with Tax knockout, the initial codon of Tax was mutated from ATG to TTG, resulting in the generation of pX1MT-M-TTG-Tax.

### Dual luciferase reporter assay

2.0 × 10^5^ HeLa S3 cells were seeded in a 24-well plate, and the next day 0.5 μg total plasmid including 10 ng pRL-TK, 0.1 μg 3’LTR-Luc and 0.4 μg control or Tax were transfected with Lipofectamine 2000 (ThermoFisher Scientific). After 24 h, the luciferase reporter assay was performed according to the manufacturer’s protocol (Promega, Madison, USA).

### Proximity labeling of the Tax protein and the HBZ RNA

miniTurboID experiments were performed as described [41]. 5 × 10⁵ HeLa S3 cells were seeded into a 6-well plate and transfected with 2 μg of Tax-miniTurboID or empty control plasmid using Lipofectamine 2000, or 1 μg of pCMV-NLS-MCP-mCherry-miniTurboID, along with 1 μg of pCMV-pre-HBZ-MS2 or empty control. After 42 hours of culture, cells were treated with 500 μM biotin (Bidepharm, China) for 6 hours before being washed with 1 × PBS and lysed for 10 minutes using 1 ml of 1 × RIPA buffer (50 mM Tris-Cl, pH 8; 150 mM NaCl; 2 mM EDTA; 1% Triton X-100; 0.1% SDS) containing a protease inhibitor cocktail (APExBIO, USA). The lysed samples were rotated for 20 minutes and centrifuged at 12,000g for 10 minutes at 4°C to remove cell debris. The supernatant was collected, and the protein concentration was measured using the Bradford protein kit (Vazyme, China).

Next, 50 μl of streptavidin agarose beads (Beyotime, China) were washed once with 1 ml of 1 × RIPA buffer and then resuspended in 1 ml of the same buffer containing 2 mg of protein lysates and incubated overnight at 4°C with rotation. The following day, the samples were centrifuged at 600g for 5 minutes, and the bead pellet was washed twice with 1 ml of 2% sodium dodecyl sulfate (SDS) solution, followed by three washes with 1 ml of Wash Buffer (50 mM Tris-HCl, pH 7.4, and 8 M urea) at room temperature for 8 minutes. Finally, the samples were resuspended in 50 μl of 1 × RIPA buffer and stored on ice.

### Data-independent acquisition-liquid chromatography-tandem mass spectrometry (DIA-LC/MS)

The protein samples were reduced with 5 mM DTT (Thermo) for 1 hour at 37°C and alkylated with 10 mM iodoacetamide (Sigma) for 45 minutes in the dark at room temperature. Proteins were precipitated using methanol/chloroform, then washed with 1 ml of methanol and resolved in 8M urea. Digestion was performed with LysC (Wako, 129–02541) for 2 hours and with trypsin (Promega, V511X) overnight, both at a 1:50 enzyme-to-protein ratio at room temperature. The digested samples were acidified with formic acid (FA; Sigma, F0507) to achieve a final concentration of 1% (final pH of 2–3), and centrifuged at 1,500 g for 15 minutes to clear precipitated urea from the peptide lysates.

Samples were desalted using C18 SepPak columns (Waters, WAT054925) and lyophilized with a SpeedVac apparatus. After reconstitution with 0.1% formic acid, 25 µg of proteome samples were separated using an EASY-nLC 1200 system with a gradient on an analytical column (180 mm × 100 μm) packed in-house with C18-AQ 1.9 μm C18 resin (Dr. Maisch GmbH, Germany). The gradient consisted of 5–28% solvent B (0.1% FA/80% ACN) over 45 minutes, at a flow rate of 250 nL/min.

MS data acquisition in DIA mode was performed on an Orbitrap Fusion Lumos, using 30 variable windows covering a mass range of 350–1500 m/z. The resolution was set to 60,000 for MS1 and 15,000 for MS2. The automatic gain control (AGC) was set to 3e6 for MS1 and 5e5 for MS2, with a maximum injection time of 100 ms for MS1 and automatic for MS2. The normalized collision energy (NCE) was set to 28%.

For data analysis, the search parameters in DIA-NN (version 1.8.1) were configured as follows: precursor false discovery rate (FDR) at 1%; mass accuracy at MS1 and MS2 set to 5 ppm and 15 ppm for LC-MS data; scan window set to 0; isotopologues and model-based re-calibration (MBR) enabled; protein inference at the gene level; heuristic protein inference enabled; quantification strategy set to Robust LC (high precision); neural network classifier with double-pass mode; and cross-run normalization turned off. The main search settings were consistent, with additional settings for in silico library generation as follows: Trypsin/P with a maximum of 1 missed cleavage; protein N-terminal M excision enabled; carbamidomethylation on C as a fixed modification; no variable modifications; peptide length from 7 to 30; precursor charge from 1 to 4; precursor m/z from 300 to 1300; and fragment m/z from 300 to 1800.

### ONT cDNA-seq and RNA-seq

For Nanopore sequencing, total RNA was treated with the Ribo-Zero rRNA Removal Kit (Illumina, USA) and then purified and concentrated using an RNA Clean Concentrator-5 column (Zymo Research, USA). cDNA libraries were generated from a mixture of 50 ng RNA and 0.5 ng Spike-in RNA Variant Control Mix E2 (Lexogen, Austria) following the Oxford Nanopore Technologies’ protocol for “DNA-PCR Sequencing”, which included 14 cycles of PCR with an elongation time of 8 minutes. ONT adapters were ligated to 650 ng of cDNA.

For conventional RNA-Seq, library preparations were conducted using a mix of 250 ng total RNA and 0.25 ng Spike-in RNA Variant Control Mix E2 (Lexogen, Austria) with the TruSeq RNA Library Prep Kit (Illumina). The ready-to-sequence libraries were quantified by qPCR using the KAPA Library Quantification Kit for Illumina Libraries (Kapa Biosystems, USA), and library profiles were evaluated using an Agilent 2100 Bioanalyzer (Agilent Technologies, USA).

### RNA extraction, RT-PCR, Tag-RT-qPCR and qPCR

RNA was extracted using the Total RNA Extraction Kit (R701, Vazyme, China), and cDNA was synthesized with the HiScript III 1st Strand cDNA Synthesis Kit (R312, Vazyme, China). For the construction of the cDNA library for RT-PCR, reverse transcription was performed using random primers or an HBZ specific RT primer. For the cDNA library for Tag-RT-qPCR, the Tag-RT primer was utilized for reverse transcription. The expression levels of the genes of interest were quantified using the QuantStudio 3 Real-Time PCR System (Thermo). The primer sequences are listed in S5 Table. The relative expression of genes was calculated using the 2^-ΔΔCt^ method with ACTB or 18S as the internal reference. The relative HBZ splicing efficiency and relative HBZ intron retention index were obtained by normalizing the relative expression of sHBZ or usHBZ to that of total HBZ.

### Quantification of HTLV-1 proviral load

Genomic DNA was isolated with the FastPure Cell/Tissue DNA Isolation Mini Kit (DC102–01, Vazyme, China). HTLV-1 proviral load was quantified by measuring HTLV-1 Gag DNA copies relative to the endogenous ACTB gene using a QuantStudio 3 Real-Time PCR System (Thermo Fisher Scientific). All primer sequences are provided in S5 Table.

### Western blot

Cells were harvested and lysed using RIPA lysis buffer (50 mM Tris-Cl, pH 8; 150 mM NaCl; 2 mM EDTA; 1% Triton X-100; 0.1% SDS), supplemented with a protease inhibitor cocktail (APExBIO, China). Cell lysates were separated by SDS-PAGE, followed by electroblotting onto polyvinylidene difluoride (PVDF) membranes and probing with specific antibodies. The Tax antibody (sc-57872) was obtained from Santa Cruz (USA), while the WDR83 antibody (27244–1-AP) and GPATCH1 antibody (D164014) were purchased from Proteintech (China) and Sangon Biotech (China), respectively. Other antibodies, including β-tubulin (T2146), Flag (T8146), HA (2367), and HRP-linked anti-rabbit IgG (7074S) and anti-mouse IgG (7076S), were purchased from Cell Signaling Technology (USA). Streptavidin-HRP was purchased from Beyotime. The WB bands were exposed using ECL luminescent solution (Sparkjade Biotechnology, China) and imaged on the Tanon 5200 Multi (Tanon Life Science, China).

### Co-IP

HeLa S3 cells were seeded at a density of 5 × 10⁵ cells/ml in a 6-well plate, and the cells were transfected with 1 μg of HA-Tax and Flag-WDR83 or Flag-GPATCH1 using Lipofectamine 2000 (Thermo). After 48 hours, total protein was extracted. For ATL2 and ATL-T, 1 × 10⁶ cells were used for total protein extraction. The samples were pre-cleared with rProtein A/G Beads 4FF (Smart-Lifesciences, China) by rotating at 4°C for 2 hours. IP was performed overnight using 5 μg of Flag or IgG antibody. rProtein A/G Beads 4FF were blocked using QuickBlock buffer (Beyotime) for 1 hour, washed, and then incubated with the protein/antibody complexes for 3 hours at 4°C with rotation. The beads were washed five times with RIPA lysis buffer (with protease inhibitors). The protein complexes were eluted by boiling for 5 minutes in 1 × SDS sample buffer and analyzed by Western blot.

### Lentivirus mediated knockdown

Lentivirus-based shRNA system was used to knock down Tax in T cell lines. Briefly, plasmids including pMD2.G (7 μg), psPAX2 (14 μg), and pLKO.1 (14 μg) were transfected with Polyethylenimine (Prime-P100, Serochem, USA) into 5.5 × 10^6^ 293T cells to produce lentivirus in a 10-cm dish. Supernatants were collected at 48 h and 72 h, filtered with a 0.45 μm filter, and then used to infect target cells. RNAs and proteins of target cells were extracted 48 hours postinfection.

### Antisense oligos (ASO)

ASOs against Tax RNA were designed and synthesized by Gencefe (Wuxi, China), and a final concentration of 50 nM was used. ASOs were transfected by Lipofectamine 2000 (ThermoFisher Scientific). The sequences of ASOs against Tax RNA are gggccttcctcaccaatgtt (#1) and gaggcagatgacaatgacca (#2). The sequence of the negative control ASO is gcgtattatagccgattaac (NC).

### WDR83 and GPATCH1 siRNA silencing

siRNAs targeting WDR83 (1: agtgcgagccgtacgatttaa, 2: gtgaacacggtgcagtttaat) and GPATCH1 (1: ggagaaattggactgaataaa, 2: acagcacccaggcagtataaa) were synthesized by Gencefe. These siRNAs were used at a final concentration of 50 nM to knock down WDR83 or GPATCH1 in HeLa S3. 48 hours post transfection, RNA was extracted for further analyses.

### HTLV-1 infection of primary CD4 T cells

Lentivirus-based system was employed to infect CD4 T cells. Briefly, plasmids including pMD2.G (7 μg), psPAX2 (14 μg) and either pX1MT-M-WT or pX1MT-M-TTG-Tax (14 μg) were transfected with Polyethylenimine into 5.5 × 10^6^ 293T cells to produce lentivirus in a 10-cm dish. Supernatants were collected at 48 h and 72 h, filtered with a 0.45 μm filter, and then used to infect primary CD4 T cells. RNAs and proteins of target cells were extracted 48 hours postinfection.

### CCK8

For transient transfection, CEMT4 cells were electroporated and then seeded into a 12-well plate. For CEMT4-sHBZ or CEMT4-Ctrl lines, 2 × 10⁵ cells were seeded in a 12-well plate and treated with 2% DMSO. At specific time points, 100 μL of the cell suspension was transferred to a 96-well plate, and 10 μL of CCK-8 reagent (Adamas Life, China) was added. The cells were then incubated in the dark in a humidified incubator at 37°C with 5% CO₂. After 3 hours, the absorbance at 450 nm was measured using a plate reader (Berthold, Germany).

### Single molecule RNA-FISH

The RNAscope RNA-FISH kit was purchased from Advanced Cell Diagnostics (ACD, USA). All procedures were performed following the protocol of the manufacturer. Cultured cells were prepared on slides by cytospin before fixation and probe hybridization. Probes used in this study are HTLV-1 HBZ intron (#472861) and HTLV-1 Tax (#472871). Slides of RNA-FISH were observed under an FV3000 fluorescent confocal microscope (Olympus).

### Immunofluorescence (IF)

HeLa S3 cells were transfected with plasmids and 48 hours after transfection, the cells were treated with 4% paraformaldehyde for 10 minutes. Following washing, the cells were incubated in PBS containing 0.3% Triton X-100 for 10 minutes, then blocked with QuickBlock buffer (Beyotime) for 30 minutes. Streptavidin-FITC (Beyotime) or anti-Flag AF488 Conjugate (Cell Signaling Technology) was added, and the cells were incubated at room temperature for 1 hour, followed by Hoechst staining. Immunofluorescence images were captured using an FV3000 fluorescent confocal microscope.

### RIP-qPCR

3 × 10⁷ cells were collected and lyzed using a cell lysis buffer (20 mM Tris-HCl (pH 7.4), 100 mM KCl, 5 mM MgCl₂, and 0.5% Triton X-100) supplemented with protease and RNase inhibitors (Beyotime). BeyoMag Protein A + G magnetic beads (Beyotime) were pre-incubated with the target antibodies. Next, antibody-bead complexes were mixed with the cell lysate and incubated overnight at 4°C in an IP buffer (50 mM Tris-HCl (pH 7.4), 150 mM NaCl, 1 mM MgCl₂, 0.05% Triton X-100, 20 mM EDTA (pH 8.0), 1 mM DTT, and 0.2 U/µL RNase inhibitor). The bead complexes were washed with wash buffer and digested with Proteinase K at 55°C for 30 minutes. Finally, RNA was extracted using the phenol-chloroform method [58]. RT-qPCR was performed to assess the enrichment of specific fragments, which was normalized to input as follows: ΔCt[normalized RIP]=(Average Ct[RIP]−(Average Ct[Input]−log2(Input Dilution Factor))), %Input = 2^ − ΔCt[normalized RIP] [59].

### Cell cycle assay

A cell cycle assay kit (Elabscience, China) was used. Briefly, 2 × 10⁵ CEMT4-sHBZ or CEMT4-Ctrl cells were treated with 2% DMSO for 48 hours. The cells were then collected, washed twice with PBS, and centrifuged at 300 g for 5 minutes. Then, the cells were resuspended in 100 µL of RNase A Reagent and incubated in a 37°C water bath for 30 minutes. Next, 400 µL of PI (50 µg/mL) was added, and the cells were further incubated for 30 minutes at room temperature, protected from light. The cells were analyzed using a NovoCyte Flow Cytometer (Agilent Technologies, USA). Data analysis was performed using FlowJo v10.0 (FlowJo, LLC).

### Cell apoptosis assay

Apoptosis was measured using the Annexin V-FITC/PI Apoptosis Kit (Elabscience) according to the manufacturer’s instructions. Briefly, 2 × 10⁵ CEMT4-sHBZ or CEMT4-Ctrl cells were treated with 2% DMSO for 48 hours. The cells were then washed three times with PBS and resuspended in 100 μL of 1 × Annexin V Binding Buffer, supplemented with 2.5 μL of Annexin V-FITC and 2.5 μL of PI. The cells were incubated at room temperature for 15 minutes in the dark. 400 μL of Annexin V Binding Buffer was added, and the cells were immediately analyzed using a NovoCyte Flow Cytometer.

### Xenograft model

4 to 5 week-old female NOD-scid IL2rγnull (NSG) mice (Hangzhou Ziyuan Experimental Animal Technology, China) were maintained in a specific pathogen-free room at the Animal Experimental Center of China Pharmaceutical University. 5.0 × 10^6^ CEMT-Ctrl or CEMT4-sHBZ cells in 50 μL of Matrigel Matrix (BD) and 50 μL of PBS were resuspend and then subcutaneously injected into mice. At the end of the experiment, mice were killed, and tumors were collected for further measurements or analyses. Tumor size was estimated by measuring the longest diameter of the entire tumor and the corresponding vertical diameter (Formula: Tumor volume (mm^3^) = 0.5 × L(length) × W^2^ (width)). All the animal experiments were approved by the Institutional Animal Care Board of China Pharmaceutical University.

### Bioinformatic analyses

Raw reads from ONT cDNA-seq were aligned to the HTLV-1 J02029 genome using minimap2 v2.26 [60]. For raw reads of RNA-seq, alignment was conducted with STAR v2.7.11a [61]. The unique alignments were extracted with Samtools v1.19 [62]. The ONT cDNA-seq alignment was subsequently corrected using the RNA-seq alignment results via Flair Corrected v2.0 [63]. Sashimi plots were generated with IGV v2.15.2 [64].

RNA-seq reads of CEMT4-Ctrl and CEMT4-sHBZ were aligned to the hg38 genome using STAR. The unique alignments were extracted with Samtools. To identify DEGs, reads were counted using HTSeq-count v0.12.3 [65], and DESeq2 v1.46.0 [66] was applied with the following thresholds: FC ≥ 2 and p-value < 0.01.

For strand-specific RNA-seq data from the ENA database (S6 Table), reads were aligned to the HTLV-1 J02029 genome using STAR. The unique alignments were extracted using Samtools. Read quantification for HBZ and Tax was performed using HTSeq-count. Host genes were analyzed using featureCounts v2.0.7 [67]. To specifically extract HTLV-1 antisense reads, we set the parameters -f 64 -F 16 and -f 128 -f 16 to identify the forward and reverse antisense reads, respectively. We then used Samtools view to extract the F2R1 (Forword in Read 2 and Reverse in Read 1) reads accordingly. Finally, we merged the extracted reads using Samtools merge. HBZ splicing junctions (SJ) were analyzed with RSeQC v5.0.1 [68], and the number of reads at canonical splice sites (GT-AG) was manually counted. Reads within the HBZ region were extracted and imported into IGV to generate a Sashimi plot.

In the proteomics analysis of Tax protein and HBZ RNA near-marker proteins, positive proteins were identified using a 1.2-fold threshold. Enrichment analysis was conducted with the ClusterProfiler v4.14.0 [69] tool, which included GO [70] annotations for molecular functions and biological processes, along with KEGG [71] pathway analysis. Enrichment scores were determined by comparing results to a hypergeometric distribution (equivalent to a one-tailed Fisher’s exact test) for each annotation group. Additionally, multiple testing correction was applied using the Benjamini-Hochberg method.

Subcellular localization analysis of potential interacting proteins was done using the Cell Atlas [47] database (https://www.proteinatlas.org/). For HBZ RNA splicing site analysis, the genomes of 314 HTLV-1 strains were obtained from the DDBJ database (https://www.ddbj.nig.ac.jp/) and aligned using MAFFT v7.511 [72] software.

### Statistical analyses

Data analysis and plotting were done using R 4.4.2 and GraphPad Prism 8. Pearson’s correlation coefficient analyzes the correlation between the two groups. A value of *P* < 0.05 was considered statistically significant. Statistical analyses were performed by using the unpaired Student t-test.

## Supporting information

S1 Fig Analysis of HBZ RNA splicing patterns, primer specificity validation, and the correlation between HBZ expression and proviral load in HTLV-1 infected cell lines. (A) Analysis of HBZ RNA splicing characteristics of Tax+ and Tax- HTLV-1 infected cell lines based on strand-specific RNA-seq data. Raw data were obtained from the ENA database (https://www.ebi.ac.uk/ena) and sample accession IDs are included in S6 Table. (B) Primer specificity was verified by agarose-gel electrophoresis in Tag-RT-qPCR and RT-qPCR assays. (C) Correlation analysis between relative proviral loads and HBZ RNA abundances using Pearson’s method.
(TIF)

S2 Fig The dual-luciferase reporter assay result demonstrates that Tax activates the HTLV-1 3’ LTR activity in HeLa S3 cells. The result is a representative of three independent experiments.
(TIF)

S3 Fig Construction and experimental validation of the Tax-miniTurboID plasmid. (A) Schematic diagrams of the miniTurboID experiment plasmid composition. (B) Immunoblot result showing successful expression of the Tax-miniTurboID fusion protein. (C) IF demonstrates the distribution of Tax-miniTurboID within the Hela S3 cells (scale bar, 10 μm). (D) PCA analysis of DIA-LC/MS data in Tax-miniTurboID experiment.
(TIF)

S4 Fig Construction and experimental validation of the HBZ pre-RNA proximity labeling plasmid. (A) Schematic diagrams of the control and HBZ pre-RNA RNA-bioID plasmid composition. (B) Immunoblot showing successful expression of the 2X MCP-mCherry-miniTurboID fusion protein. (C) IF demonstrates the distribution of 2X MCP-mCherry-miniTurboID and biotinylated proteins in Hela S3 cells (scale bar, 10 μm).
(TIF)

S5 Fig sHBZ promotes the proliferation of CEMT4 cells in vitro. (A) qPCR result of sHBZ expression in CEMT4-sHBZ stable transfectant and HTLV-1 infected ED cell line. (B) The promoting effect of sHBZ on cell proliferation in stable transfectant of CEMT4. (C) KEGG enrichment analysis of host genes downregulated by sHBZ in CEMT4. (D) Flow cytometry results showing sHBZ exhibits anti-apoptotic effect upon 2% DMSO induced apoptosis. (E) Statistical analysis of the data presented in (D). (F) Flow cytometry results showing sHBZ promotes cell cycle progression upon 2% DMSO induced cell cycle arrest. (G) Statistical analysis of the data presented in (F). ^**^P < 0.01, ^***^P < 0.001. The results are representatives of three independent experiments.
(TIF)

S1 Table Identification of proximity labeling proteins of Tax via DIA-LC/MS. (XLSX)

S2 Table GO (Biological progress) enrichment of Tax-interacting proteins. (XLSX)

S3 Table KEGG Pathway enrichment of Tax-interacting proteins. (XLSX)

S4 Table Identification of proximity labeling proteins of HBZ pre-RNA via DIA-LC/MS. (XLSX)

S5 Table List of primer sequences related to experimental procedures. (XLSX)

S6 Table The source of RNA-seq data. (XLSX)

## References

[1] Mui UN, Haley CT, Tyring SK. Viral Oncology: Molecular Biology and Pathogenesis. J Clin Med. 2017;6(12):111. doi: 10.3390/jcm6120111 29186062 PMC5742800

[2] Marusawa H, Uemoto S, Hijikata M, Ueda Y, Tanaka K, Shimotohno K, et al. Latent hepatitis B virus infection in healthy individuals with antibodies to hepatitis B core antigen. Hepatology. 2000;31(2):488–95. doi: 10.1002/hep.510310232 10655275

[3] Revill PA, Locarnini SA. New perspectives on the hepatitis B virus life cycle in the human liver. J Clin Invest. 2016;126(3):833–6. doi: 10.1172/JCI86650 26901815 PMC4767364

[4] Indolfi G, Mangone G, Bartolini E, Moriondo M, Azzari C, Resti M. Hepatitis C viraemia after apparent spontaneous clearance in a vertically infected child. Lancet. 2016;387(10031):1967–8. doi: 10.1016/S0140-6736(16)00085-4 27203660

[5] Yu Y, Wang SE, Hayward GS. The KSHV immediate-early transcription factor RTA encodes ubiquitin E3 ligase activity that targets IRF7 for proteosome-mediated degradation. Immunity. 2005;22(1):59–70. doi: 10.1016/j.immuni.2004.11.011 15664159

[6] Schulz TF, Freise A, Stein SC. Kaposi sarcoma-associated herpesvirus latency-associated nuclear antigen: more than a key mediator of viral persistence. Curr Opin Virol. 2023;61:101336. doi: 10.1016/j.coviro.2023.101336 37331160

[7] Gessain A, Cassar O. Epidemiological Aspects and World Distribution of HTLV-1 Infection. Front Microbiol. 2012;3:388. doi: 10.3389/fmicb.2012.00388 23162541 PMC3498738

[8] Matsuoka M, Jeang K-T. Human T-cell leukaemia virus type 1 (HTLV-1) infectivity and cellular transformation. Nat Rev Cancer. 2007;7(4):270–80. doi: 10.1038/nrc2111 17384582

[9] Yamano Y, Sato T. Clinical pathophysiology of human T-lymphotropic virus-type 1-associated myelopathy/tropical spastic paraparesis. Front Microbiol. 2012;3:389. doi: 10.3389/fmicb.2012.00389 23162542 PMC3494083

[10] Zuo X, Zhou R, Yang S, Ma G. HTLV-1 persistent infection and ATLL oncogenesis. J Med Virol. 2023;95(1):e28424. doi: 10.1002/jmv.28424 36546414

[11] Arnold J, Zimmerman B, Li M, Lairmore MD, Green PL. Human T-cell leukemia virus type-1 antisense-encoded gene, Hbz, promotes T-lymphocyte proliferation. Blood. 2008;112(9):3788–97. doi: 10.1182/blood-2008-04-154286 18689544 PMC2572803

[12] Arnold J, Yamamoto B, Li M, Phipps AJ, Younis I, Lairmore MD, et al. Enhancement of infectivity and persistence in vivo by HBZ, a natural antisense coded protein of HTLV-1. Blood. 2006;107(10):3976–82. doi: 10.1182/blood-2005-11-4551 16424388 PMC1895283

[13] Bangham CRM. HTLV-1 persistence and the oncogenesis of adult T-cell leukemia/lymphoma. Blood. 2023;141(19):2299–306. doi: 10.1182/blood.2022019332 36800643 PMC10646791

[14] Kulkarni A, Bangham CRM. HTLV-1: Regulating the Balance Between Proviral Latency and Reactivation. Front Microbiol. 2018;9:449. doi: 10.3389/fmicb.2018.00449 29615991 PMC5867303

[15] Maksimova V, Wilkie T, Smith S, Phelps C, Melvin C, Yu L, et al. HTLV-1 Hbz protein, but not hbz mRNA secondary structure, is critical for viral persistence and disease development. PLoS Pathog. 2023;19(6):e1011459. doi: 10.1371/journal.ppat.1011459 37327244 PMC10309998

[16] Ma G, Yasunaga J-I, Matsuoka M. Multifaceted functions and roles of HBZ in HTLV-1 pathogenesis. Retrovirology. 2016;13:16. doi: 10.1186/s12977-016-0249-x 26979059 PMC4793531

[17] Murata K, Hayashibara T, Sugahara K, Uemura A, Yamaguchi T, Harasawa H, et al. A novel alternative splicing isoform of human T-cell leukemia virus type 1 bZIP factor (HBZ-SI) targets distinct subnuclear localization. J Virol. 2006;80(5):2495–505. doi: 10.1128/JVI.80.5.2495-2505.2006 16474156 PMC1395368

[18] Cavanagh M-H, Landry S, Audet B, Arpin-André C, Hivin P, Paré M-E, et al. HTLV-I antisense transcripts initiating in the 3’LTR are alternatively spliced and polyadenylated. Retrovirology. 2006;3:15. doi: 10.1186/1742-4690-3-15 16512901 PMC1459196

[19] Satou Y, Yasunaga J, Yoshida M, Matsuoka M. HTLV-I basic leucine zipper factor gene mRNA supports proliferation of adult T cell leukemia cells. Proc Natl Acad Sci U S A. 2006;103(3):720–5. doi: 10.1073/pnas.0507631103 16407133 PMC1334651

[20] Mohanty S, Harhaj EW. Mechanisms of Oncogenesis by HTLV-1 Tax. Pathogens. 2020;9(7):543. doi: 10.3390/pathogens9070543 32645846 PMC7399876

[21] Zhao T, Satou Y, Sugata K, Miyazato P, Green PL, Imamura T, et al. HTLV-1 bZIP factor enhances TGF-β signaling through p300 coactivator. Blood. 2011;118(7):1865–76. doi: 10.1182/blood-2010-12-326199 21705495 PMC3158717

[22] Bellon M, Nicot C. HTLV-1 Tax Tug-of-War: Cellular Senescence and Death or Cellular Transformation. Pathogens. 2024;13(1):87. doi: 10.3390/pathogens13010087 38276160 PMC10820833

[23] Giam C-Z, Semmes OJ. HTLV-1 Infection and Adult T-Cell Leukemia/Lymphoma-A Tale of Two Proteins: Tax and HBZ. Viruses. 2016;8(6):161. doi: 10.3390/v8060161 27322308 PMC4926181

[24] Belrose G, Gross A, Olindo S, Lézin A, Dueymes M, Komla-Soukha I, et al. Effects of valproate on Tax and HBZ expression in HTLV-1 and HAM/TSP T lymphocytes. Blood. 2011;118(9):2483–91. doi: 10.1182/blood-2010-11-321364 21505188

[25] Uppal T, Banerjee S, Sun Z, Verma SC, Robertson ES. KSHV LANA--the master regulator of KSHV latency. Viruses. 2014;6(12):4961–98. doi: 10.3390/v6124961 25514370 PMC4276939

[26] Gaudray G, Gachon F, Basbous J, Biard-Piechaczyk M, Devaux C, Mesnard J-M. The complementary strand of the human T-cell leukemia virus type 1 RNA genome encodes a bZIP transcription factor that down-regulates viral transcription. J Virol. 2002;76(24):12813–22. doi: 10.1128/jvi.76.24.12813-12822.2002 12438606 PMC136662

[27] Nozuma S, Kubota R, Jacobson S. Human T-lymphotropic virus type 1 (HTLV-1) and cellular immune response in HTLV-1-associated myelopathy/tropical spastic paraparesis. J Neurovirol. 2020;26(5):652–63. doi: 10.1007/s13365-020-00881-w 32705480 PMC7532128

[28] Dvinge H, Kim E, Abdel-Wahab O, Bradley RK. RNA splicing factors as oncoproteins and tumour suppressors. Nat Rev Cancer. 2016;16(7):413–30. doi: 10.1038/nrc.2016.51 27282250 PMC5094465

[29] Ajiro M, Zheng Z-M. Oncogenes and RNA splicing of human tumor viruses. Emerg Microbes Infect. 2014;3(9):e63. doi: 10.1038/emi.2014.62 26038756 PMC4185361

[30] Arora R, Chang Y, Moore PS. MCV and Merkel cell carcinoma: a molecular success story. Curr Opin Virol. 2012;2(4):489–98. doi: 10.1016/j.coviro.2012.05.007 22710026 PMC3422445

[31] Chua MA, Schmid S, Perez JT, Langlois RA, Tenoever BR. Influenza A virus utilizes suboptimal splicing to coordinate the timing of infection. Cell Rep. 2013;3(1):23–9. doi: 10.1016/j.celrep.2012.12.010 23333274 PMC3563938

[32] Ma G, Yasunaga J-I, Shimura K, Takemoto K, Watanabe M, Amano M, et al. Human retroviral antisense mRNAs are retained in the nuclei of infected cells for viral persistence. Proc Natl Acad Sci U S A. 2021;118(17):e2014783118. doi: 10.1073/pnas.2014783118 33875584 PMC8092383

[33] Matsuo M, Ueno T, Monde K, Sugata K, Tan BJY, Rahman A, et al. Identification and characterization of a novel enhancer in the HTLV-1 proviral genome. Nat Commun. 2022;13(1):2405. doi: 10.1038/s41467-022-30029-9 35504920 PMC9065021

[34] Scotto L, Kinahan C, Douglass E, Deng C, Safari M, Casadei B, et al. Targeting the T-Cell Lymphoma Epigenome Induces Cell Death, Cancer Testes Antigens, Immune-Modulatory Signaling Pathways. Mol Cancer Ther. 2021;20(8):1422–30. doi: 10.1158/1535-7163.MCT-20-0377 34108263 PMC8941846

[35] Shallak M, Alberio T, Fasano M, Monti M, Iacobucci I, Ladet J, et al. The endogenous HBZ interactome in ATL leukemic cells reveals an unprecedented complexity of host interacting partners involved in RNA splicing. Front Immunol. 2022;13:939863. doi: 10.3389/fimmu.2022.939863 35979358 PMC9376625

[36] Kobayashi-Ishihara M, Yamagishi M, Hara T, Matsuda Y, Takahashi R, Miyake A, et al. HIV-1-encoded antisense RNA suppresses viral replication for a prolonged period. Retrovirology. 2012;9:38. doi: 10.1186/1742-4690-9-38 22569184 PMC3410806

[37] Yoshida M, Satou Y, Yasunaga J-I, Fujisawa J-I, Matsuoka M. Transcriptional control of spliced and unspliced human T-cell leukemia virus type 1 bZIP factor (HBZ) gene. J Virol. 2008;82(19):9359–68. doi: 10.1128/JVI.00242-08 18653454 PMC2546946

[38] Byrne A, Beaudin AE, Olsen HE, Jain M, Cole C, Palmer T, et al. Nanopore long-read RNAseq reveals widespread transcriptional variation among the surface receptors of individual B cells. Nat Commun. 2017;8:16027. doi: 10.1038/ncomms16027 28722025 PMC5524981

[39] Ma G, Yasunaga J-I, Shimura K, Takemoto K, Watanabe M, Amano M, et al. Human retroviral antisense mRNAs are retained in the nuclei of infected cells for viral persistence. Proc Natl Acad Sci U S A. 2021;118(17):e2014783118. doi: 10.1073/pnas.2014783118 33875584 PMC8092383

[40] Bentley DL. Coupling mRNA processing with transcription in time and space. Nat Rev Genet. 2014;15(3):163–75. doi: 10.1038/nrg3662 24514444 PMC4304646

[41] Branon TC, Bosch JA, Sanchez AD, Udeshi ND, Svinkina T, Carr SA, et al. Efficient proximity labeling in living cells and organisms with TurboID. Nat Biotechnol. 2018;36(9):880–7. doi: 10.1038/nbt.4201 30125270 PMC6126969

[42] Milione RR, Schell B-B, Douglas CJ, Seath CP. Creative approaches using proximity labeling to gain new biological insights. Trends Biochem Sci. 2024;49(3):224–35. doi: 10.1016/j.tibs.2023.12.005 38160064 PMC10939868

[43] Goren I, Semmes OJ, Jeang KT, Moelling K. The amino terminus of Tax is required for interaction with the cyclic AMP response element binding protein. J Virol. 1995;69(9):5806–11. doi: 10.1128/JVI.69.9.5806-5811.1995 7637025 PMC189446

[44] Kwok RP, Laurance ME, Lundblad JR, Goldman PS, Shih H, Connor LM, et al. Control of cAMP-regulated enhancers by the viral transactivator Tax through CREB and the co-activator CBP. Nature. 1996;380(6575):642–6. doi: 10.1038/380642a0 8602268

[45] Ameur LB, Marie P, Thenoz M, Giraud G, Combe E, Claude J-B, et al. Intragenic recruitment of NF-κB drives splicing modifications upon activation by the oncogene Tax of HTLV-1. Nat Commun. 2020;11(1):3045. doi: 10.1038/s41467-020-16853-x 32546717 PMC7298006

[46] Burton M, Upadhyaya CD, Maier B, Hope TJ, Semmes OJ. Human T-cell leukemia virus type 1 Tax shuttles between functionally discrete subcellular targets. J Virol. 2000;74(5):2351–64. doi: 10.1128/jvi.74.5.2351-2364.2000 10666266 PMC111717

[47] Thul PJ, Åkesson L, Wiking M, Mahdessian D, Geladaki A, Ait Blal H, et al. A subcellular map of the human proteome. Science. 2017;356(6340):eaal3321. doi: 10.1126/science.aal3321 28495876

[48] Mukherjee J, Hermesh O, Eliscovich C, Nalpas N, Franz-Wachtel M, Maček B, et al. β-Actin mRNA interactome mapping by proximity biotinylation. Proc Natl Acad Sci U S A. 2019;116(26):12863–72. doi: 10.1073/pnas.1820737116 31189591 PMC6600913

[49] Sales-Lee J, Perry DS, Bowser BA, Diedrich JK, Rao B, Beusch I, et al. Coupling of spliceosome complexity to intron diversity. Curr Biol. 2021;31(22):4898-4910.e4. doi: 10.1016/j.cub.2021.09.004 34555349 PMC8967684

[50] Rosewick N, Durkin K, Artesi M, Marçais A, Hahaut V, Griebel P, et al. Cis-perturbation of cancer drivers by the HTLV-1/BLV proviruses is an early determinant of leukemogenesis. Nat Commun. 2017;8:15264. doi: 10.1038/ncomms15264 28534499 PMC5457497

[51] Liu W, You J. Molecular Mechanisms of Merkel Cell Polyomavirus Transformation and Replication. Annu Rev Virol. 2020;7(1):289–307. doi: 10.1146/annurev-virology-011720-121757 32603631 PMC7811827

[52] Koiwa T, Hamano-Usami A, Ishida T, Okayama A, Yamaguchi K, Kamihira S, et al. 5’-long terminal repeat-selective CpG methylation of latent human T-cell leukemia virus type 1 provirus in vitro and in vivo. J Virol. 2002;76(18):9389–97. doi: 10.1128/jvi.76.18.9389-9397.2002 12186921 PMC136445

[53] Satou Y, Utsunomiya A, Tanabe J, Nakagawa M, Nosaka K, Matsuoka M. HTLV-1 modulates the frequency and phenotype of FoxP3+CD4+ T cells in virus-infected individuals. Retrovirology. 2012;9:46. doi: 10.1186/1742-4690-9-46 22647666 PMC3403885

[54] Hleihel R, Skayneh H, de Thé H, Hermine O, Bazarbachi A. Primary cells from patients with adult T cell leukemia/lymphoma depend on HTLV-1 Tax expression for NF-κB activation and survival. Blood Cancer J. 2023;13(1):67. doi: 10.1038/s41408-023-00841-7 37137914 PMC10156663

[55] Mahgoub M, Yasunaga J-I, Iwami S, Nakaoka S, Koizumi Y, Shimura K, et al. Sporadic on/off switching of HTLV-1 Tax expression is crucial to maintain the whole population of virus-induced leukemic cells. Proc Natl Acad Sci U S A. 2018;115(6):E1269–78. doi: 10.1073/pnas.1715724115 29358408 PMC5819419

[56] Ameur LB, Marie P, Thenoz M, Giraud G, Combe E, Claude J-B, et al. Intragenic recruitment of NF-κB drives splicing modifications upon activation by the oncogene Tax of HTLV-1. Nat Commun. 2020;11(1):3045. doi: 10.1038/s41467-020-16853-x 32546717 PMC7298006

[57] Vandermeulen C, O’Grady T, Wayet J, Galvan B, Maseko S, Cherkaoui M, et al. The HTLV-1 viral oncoproteins Tax and HBZ reprogram the cellular mRNA splicing landscape. PLoS Pathog. 2021;17(9):e1009919. doi: 10.1371/journal.ppat.1009919 34543356 PMC8483338

[58] Chomczynski P, Sacchi N. The single-step method of RNA isolation by acid guanidinium thiocyanate-phenol-chloroform extraction: twenty-something years on. Nat Protoc. 2006;1(2):581–5. doi: 10.1038/nprot.2006.83 17406285

[59] Gagliardi M, Matarazzo MR. RIP: RNA Immunoprecipitation. Methods Mol Biol. 2016;1480:73–86. doi: 10.1007/978-1-4939-6380-5_7 27659976

[60] Li H. New strategies to improve minimap2 alignment accuracy. Bioinformatics. 2021;37(23):4572–4. doi: 10.1093/bioinformatics/btab705 34623391 PMC8652018

[61] Dobin A, Davis CA, Schlesinger F, Drenkow J, Zaleski C, Jha S, et al. STAR: ultrafast universal RNA-seq aligner. Bioinformatics. 2013;29(1):15–21. doi: 10.1093/bioinformatics/bts635 23104886 PMC3530905

[62] Danecek P, Bonfield JK, Liddle J, Marshall J, Ohan V, Pollard MO, et al. Twelve years of SAMtools and BCFtools. GigaScience. 2021;10(2). doi: 10.1093/gigascience/giab008 PMC7931819 33590861

[63] Tang AD, Soulette CM, van Baren MJ, Hart K, Hrabeta-Robinson E, Wu CJ, et al. Full-length transcript characterization of SF3B1 mutation in chronic lymphocytic leukemia reveals downregulation of retained introns. Nat Commun. 2020;11(1):1438. doi: 10.1038/s41467-020-15171-6 32188845 PMC7080807

[64] Robinson JT, Thorvaldsdóttir H, Winckler W, Guttman M, Lander ES, Getz G, et al. Integrative genomics viewer. Nat Biotechnol. 2011;29(1):24–6. doi: 10.1038/nbt.1754 21221095 PMC3346182

[65] Putri GH, Anders S, Pyl PT, Pimanda JE, Zanini F. Analysing high-throughput sequencing data in Python with HTSeq 2.0. Bioinformatics. 2022;38(10):2943–5. doi: 10.1093/bioinformatics/btac166 35561197 PMC9113351

[66] Love MI, Huber W, Anders S. Moderated estimation of fold change and dispersion for RNA-seq data with DESeq2. Genome Biol. 2014;15(12):550. doi: 10.1186/s13059-014-0550-8 25516281 PMC4302049

[67] Liao Y, Smyth GK, Shi W. featureCounts: an efficient general purpose program for assigning sequence reads to genomic features. Bioinformatics. 2014;30(7):923–30. doi: 10.1093/bioinformatics/btt656 24227677

[68] Wang L, Wang S, Li W. RSeQC: quality control of RNA-seq experiments. Bioinformatics. 2012;28(16):2184–5. doi: 10.1093/bioinformatics/bts356 22743226

[69] Wu T, Hu E, Xu S, Chen M, Guo P, Dai Z, et al. clusterProfiler 4.0: A universal enrichment tool for interpreting omics data. Innovation (Camb). 2021;2(3):100141. doi: 10.1016/j.xinn.2021.100141 34557778 PMC8454663

[70] Ashburner M, Ball CA, Blake JA, Botstein D, Butler H, Cherry JM, et al. Gene ontology: tool for the unification of biology. The Gene Ontology Consortium. Nat Genet. 2000;25(1):25–9. doi: 10.1038/75556 10802651 PMC3037419

[71] Kanehisa M, Furumichi M, Sato Y, Matsuura Y, Ishiguro-Watanabe M. KEGG: biological systems database as a model of the real world. Nucleic Acids Res. 2024;53(D1):D672–7. doi: 10.1093/nar/gkae909 39417505 PMC11701520

[72] Rozewicki J, Li S, Amada KM, Standley DM, Katoh K. MAFFT-DASH: integrated protein sequence and structural alignment. Nucleic Acids Res. 2019;47(W1):W5–10. doi: 10.1093/nar/gkz342 31062021 PMC6602451

