# Supplementary figures and images for: Splicing-dependent restriction of the HBZ gene by Tax underlies biphasic HTLV-1 infection
Source: PLoS Pathog. 2025 Jul 28;21(7):e1013381. doi: 10.1371/journal.ppat.1013381 (PMC12313066; doi:10.1371/journal.ppat.1013381)

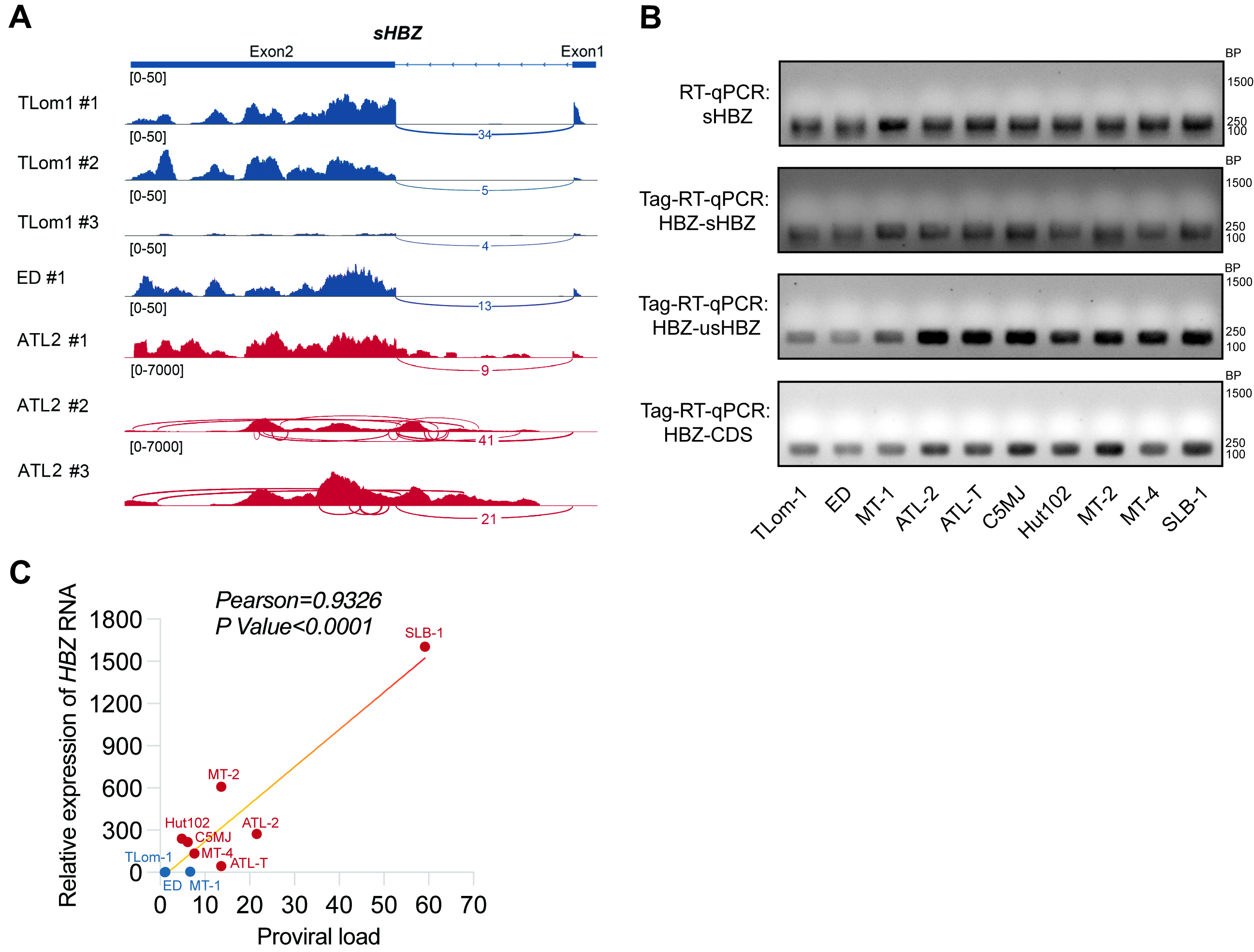

Supplement: S1 Fig — (A) Analysis of HBZ RNA splicing characteristics of Tax+ and Tax- HTLV-1 infected cell lines based on strand-specific RNA-seq data. Raw data were obtained from the ENA database (https://www.ebi.ac.uk/ena) and sample accession IDs are included in S6 Table. (B) Primer specificity was verified by agarose-gel electrophoresis in Tag-RT-qPCR and RT-qPCR assays. (C) Correlation analysis between relative proviral loads and HBZ RNA abundances using Pearson’s method. (TIF) [file ppat.1013381.s001.tif]

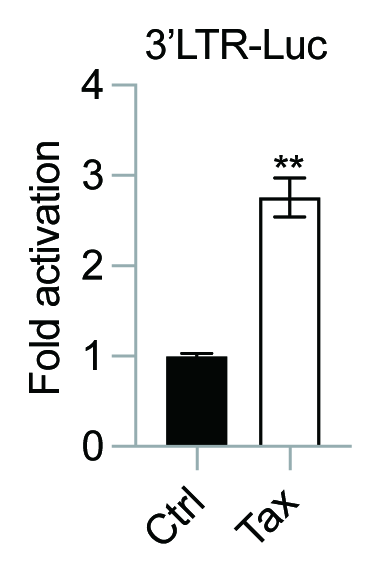

Supplement: S2 Fig — The result is a representative of three independent experiments. (TIF) [file ppat.1013381.s002.tif]

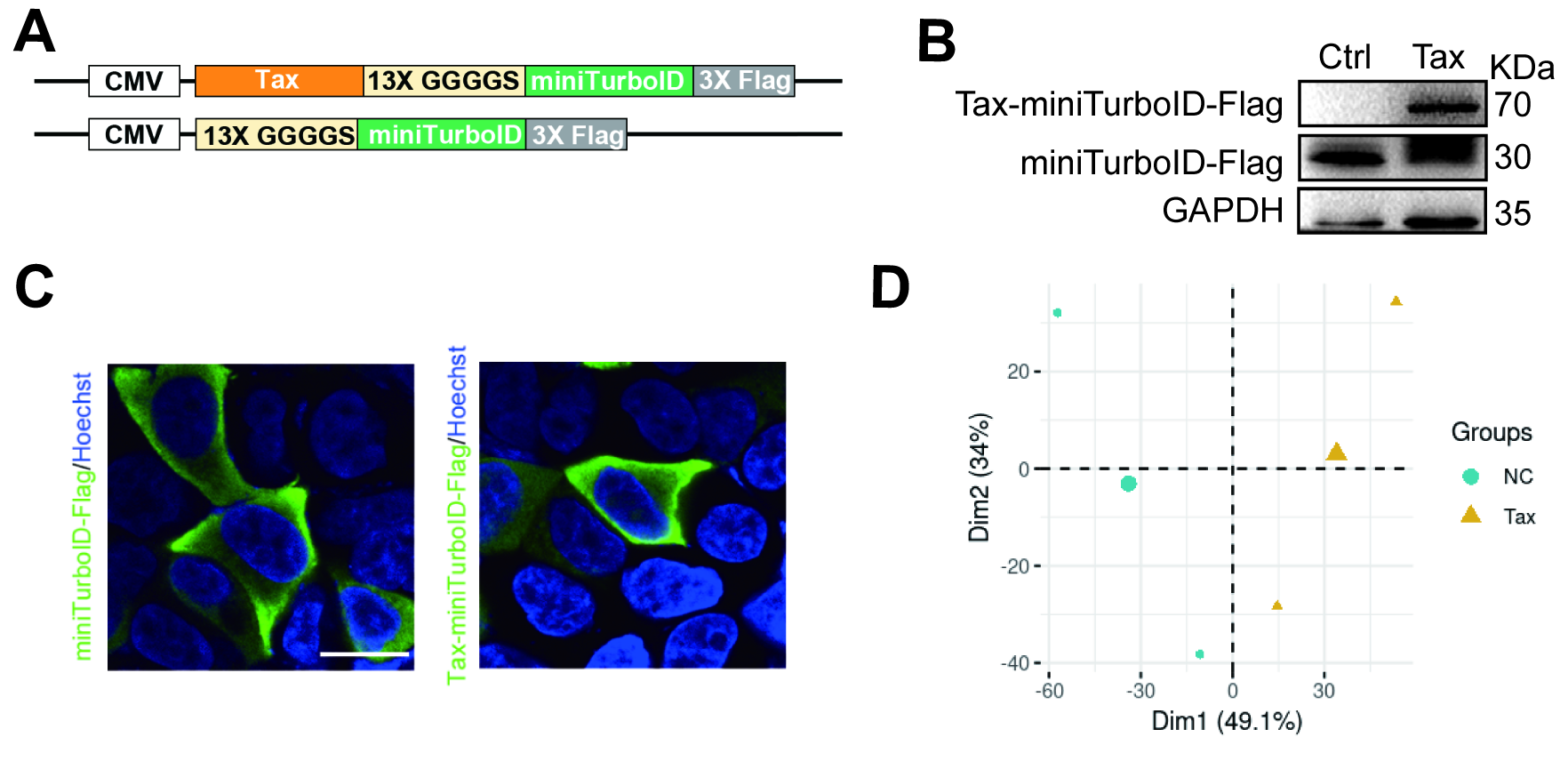

Supplement: S3 Fig — (A) Schematic diagrams of the miniTurboID experiment plasmid composition. (B) Immunoblot result showing successful expression of the Tax-miniTurboID fusion protein. (C) IF demonstrates the distribution of Tax-miniTurboID within the Hela S3 cells (scale bar, 10 μm). (D) PCA analysis of DIA-LC/MS data in Tax-miniTurboID experiment. (TIF) [file ppat.1013381.s003.tif]

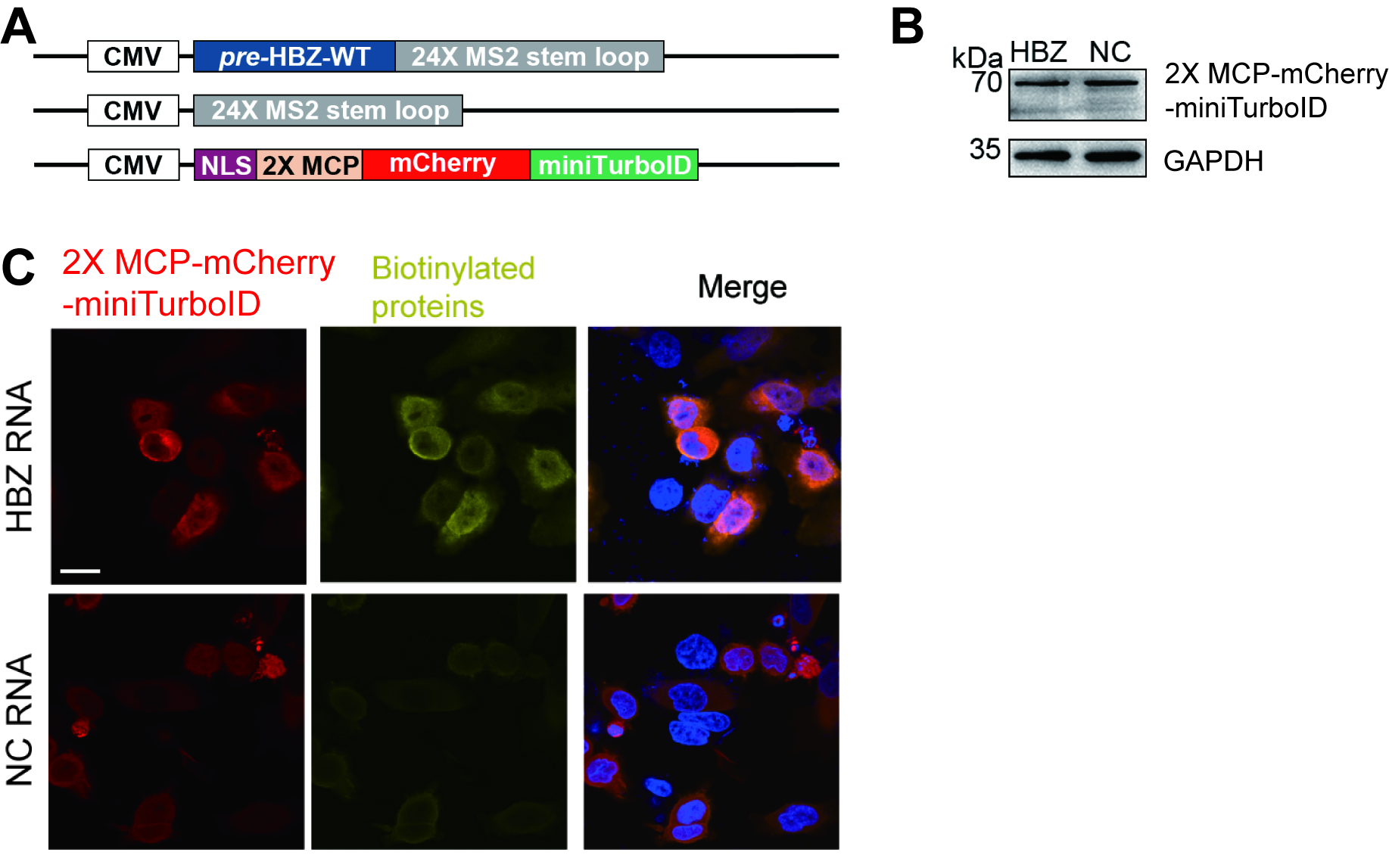

Supplement: S4 Fig — (A) Schematic diagrams of the control and HBZ pre-RNA RNA-bioID plasmid composition. (B) Immunoblot showing successful expression of the 2X MCP-mCherry-miniTurboID fusion protein. (C) IF demonstrates the distribution of 2X MCP-mCherry-miniTurboID and biotinylated proteins in Hela S3 cells (scale bar, 10 μm). (TIF) [file ppat.1013381.s004.tif]

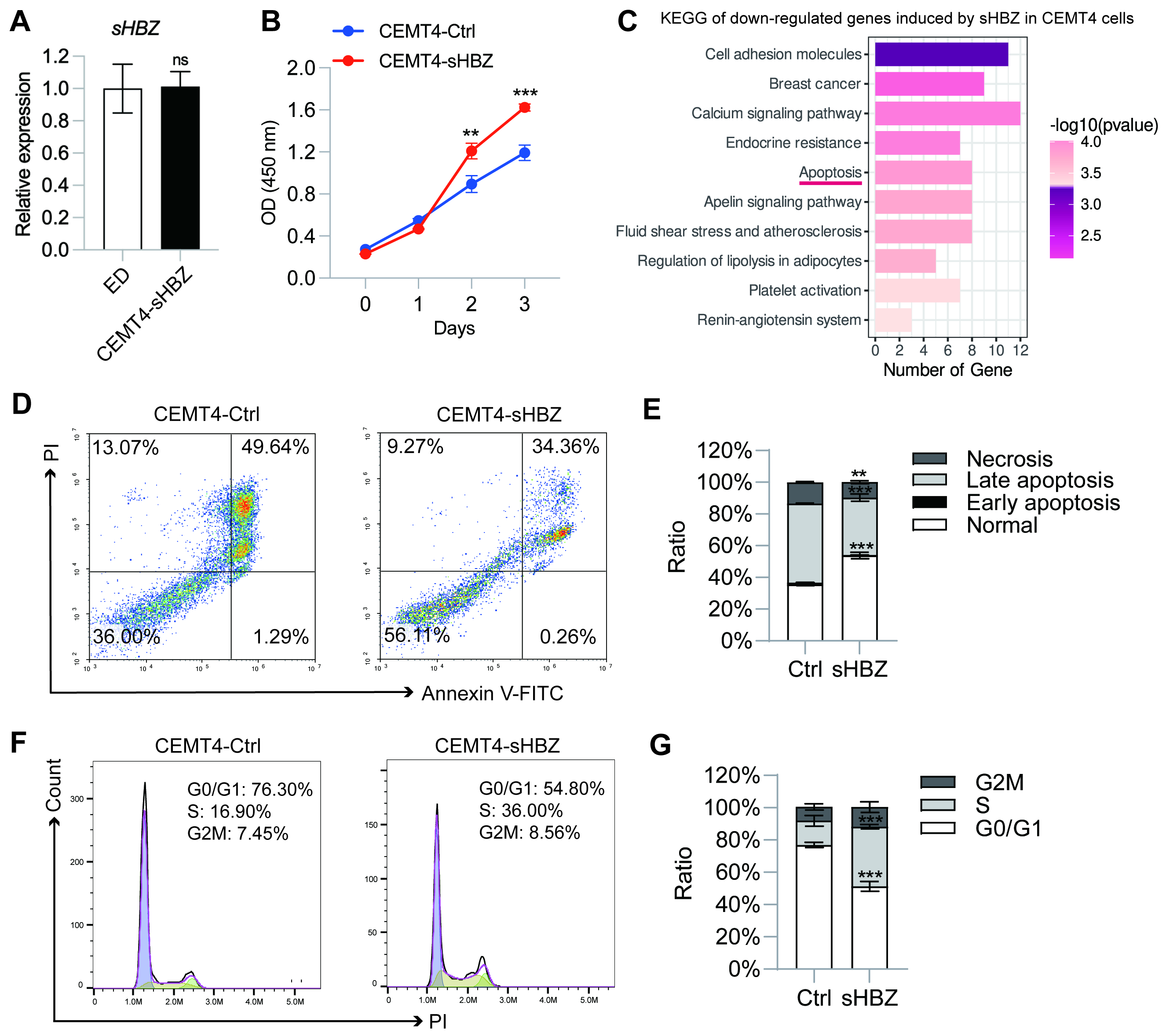

Supplement: S5 Fig — (A) qPCR result of sHBZ expression in CEMT4-sHBZ stable transfectant and HTLV-1 infected ED cell line. (B) The promoting effect of sHBZ on cell proliferation in stable transfectant of CEMT4. (C) KEGG enrichment analysis of host genes downregulated by sHBZ in CEMT4. (D) Flow cytometry results showing sHBZ exhibits anti-apoptotic effect upon 2% DMSO induced apoptosis. (E) Statistical analysis of the data presented in (D). (F) Flow cytometry results showing sHBZ promotes cell cycle progression upon 2% DMSO induced cell cycle arrest. (G) Statistical analysis of the data presented in (F). **P < 0.01, ***P < 0.001. The results are representatives of three independent experiments. (TIF) [file ppat.1013381.s005.tif]
